# Distinctive mutational spectrum and karyotype disruption in long-term cisplatin-treated urothelial carcinoma cell lines

**DOI:** 10.1038/s41598-019-50891-w

**Published:** 2019-10-09

**Authors:** Margaretha A. Skowron, Patrick Petzsch, Karin Hardt, Nicholas Wagner, Manfred Beier, Stefanie Stepanow, Matthias Drechsler, Harald Rieder, Karl Köhrer, Günter Niegisch, Michèle J. Hoffmann, Wolfgang A. Schulz

**Affiliations:** 1 Department of Urology, Medical Faculty, Heinrich Heine University Düsseldorf, Düsseldorf, Germany; 2 Biological and Medical Research Center (BMFZ), Heinrich Heine University Düsseldorf, Düsseldorf, Germany; 3 Institute for Human Genetics, Medical Faculty, Heinrich Heine University Düsseldorf, Düsseldorf, Germany

**Keywords:** Cancer genomics, Cancer genomics

## Abstract

The DNA-damaging compound cisplatin is broadly employed for cancer chemotherapy. The mutagenic effects of cisplatin on cancer cell genomes are poorly studied and might even contribute to drug resistance. We have therefore analyzed mutations and chromosomal alterations in four cisplatin-resistant bladder cancer cell lines (LTTs) by whole-exome-sequencing and array-CGH. 720–7479 genes in the LTTs contained point mutations, with a characteristic mutational signature. Only 53 genes were mutated in all LTTs, including the presumed cisplatin exporter *ATP7B*. Chromosomal alterations were characterized by segmented deletions and gains leading to severely altered karyotypes. The few chromosomal changes shared among LTTs included gains involving the anti-apoptotic *BCL2L1* gene and losses involving the NRF2 regulator *KEAP1*. Overall, the extent of genomic changes paralleled cisplatin treatment concentrations. In conclusion, bladder cancer cell lines selected for cisplatin-resistance contain abundant and characteristic drug-induced genomic changes. Cisplatin treatment may therefore generate novel tumor genomes during patient treatment.

## Introduction

Cisplatin (cis-diamminedichloroplatinum, Pt(NH_3_)_2_Cl_2_) is used as a cytotoxic chemotherapeutic compound in the treatment of various cancers. It is particularly efficacious in testicular germ cell cancers, whereas its efficacy in other cancer types, such as ovarian or urinary bladder cancer, is often limited by inherent or acquired resistance^1–3^ caused by multifaceted mechanisms^4–9^.

Following activation in the cell by aquation, cisplatin forms monoadducts with guanine bases in DNA which react with adjacent purine bases to form intrastrand crosslinks, namely about 65% *cis*-Pt(NH_3_)_2_-d(GpG), 25% *cis*-Pt(NH_3_)_2_-d(ApG) 1,2-intrastrand adducts and 5–10% 1,3-intrastrand adducts^10–12^. In addition, a small, but relevant amount of interstrand crosslinks is created. Intrastrand crosslinks are repaired by nucleotide excision repair (NER) or may be bypassed by translesion synthesis during replication. Resolving interstrand crosslinks requires the crosslink-homologous repair system. The various types of DNA damage caused directly or indirectly by cisplatin may thus lead to base mutations or even double-strand breaks and chromosomal instability. Very recently, a specific mutational signature for cisplatin was deduced from treated mammary and hepatic tumor cells and likewise detected in human tumor samples treated with cisplatin^13^.

Many mechanisms are involved in the resistance of cancer cells to cisplatin treatment, including changes in transport and metabolism of the drug, altered DNA repair and checkpoint signaling as well as evasion of apoptosis^9,14^. These mechanisms have been studied in depth in many cancer types, especially in cell line models. A less explored issue is which genomic changes are elicited by cisplatin in cancer cells that acquire resistance to the DNA-damaging drug, and whether and to which extent these contribute to the various mechanisms of resistance.

We have previously generated cisplatin-resistant variants, named LTTs (for long-term treated), from four different bladder cancer cell lines by repeated treatment with increasing concentrations of cisplatin. In the initial cell lines IC_50_ values for 72 h cisplatin treatment, as determined by standard viability assays, ranged from 1.9 to 12 µM, but increased in the respective cisplatin-resistant variants to 10 to 210 µM, yielding resistance indices of 5–17 fold^7^. They are maintained with regular cisplatin treatment, which would be lethal for the parental cells, but from which the LTTs recover within a few days. As described in detail in previous publications, the LTTs presented with a broad spectrum of resistance mechanisms, each to various extents, prominently increased cisplatin detoxification, driven partly by activation of NRF2 signaling^8^, which increases glutathione biosynthesis and the MRP2 glutathione conjugate exporter, among others, and decreased apoptosis with higher expression of anti-apoptotic factors like survivin^7^. The LTTs are not enriched for bladder cancer stem cell-like populations, but canonical WNT signaling is partly activated and some markers of epithelial-mesenchymal transition are increased^5^.

Here, we report on the genomic differences between the parental RT-112, J82, 253J, and T-24 cell lines and their cisplatin-resistant LTT variants, as determined by whole exome sequencing (WES), array comparative genomic hybridization (aCGH) and karyotyping. Based on this data, we addressed several questions. First, to which extent does cisplatin induce point mutations in bladder cancer cell lines? Second, do these mutations conform to the signature proposed by recent studies? Third, is there evidence for induction of chromosomal alterations by cisplatin? Fourth, are mutations and chromosomal alterations gained during initial selection or accrued gradually? Fifth, is it possible to identify genomic changes accounting for the observed mechanisms of resistance in the LTTs?

## Results

### Point mutations

**Figure 1:**
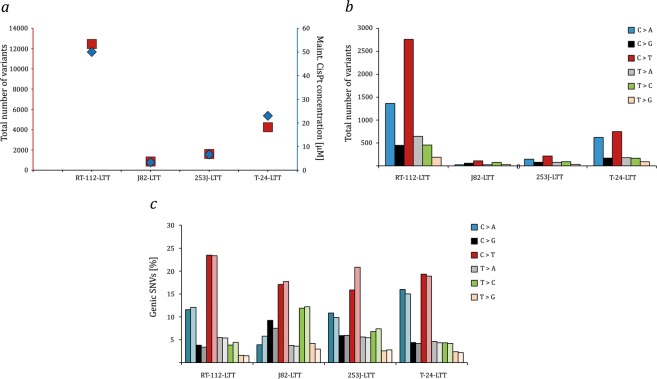
Characterization of genetic alterations in LTT lines. (**a**) Correlation between the total number of nucleotide variants and maintenance cisplatin concentration in RT-112-LTT, J82-LTT, 253J-LTT, and T-24-LTT. (**b**) Total number of exchanges for the six possible types of substitutions for each LTT subline. (**c**) Total number of exchanges for the twelve types of substitutions for each LTT subline; color codes as in (**b**). Lighter corresponding color shades denote substitutions on the nontranscribed strand.

**Figure 2:**
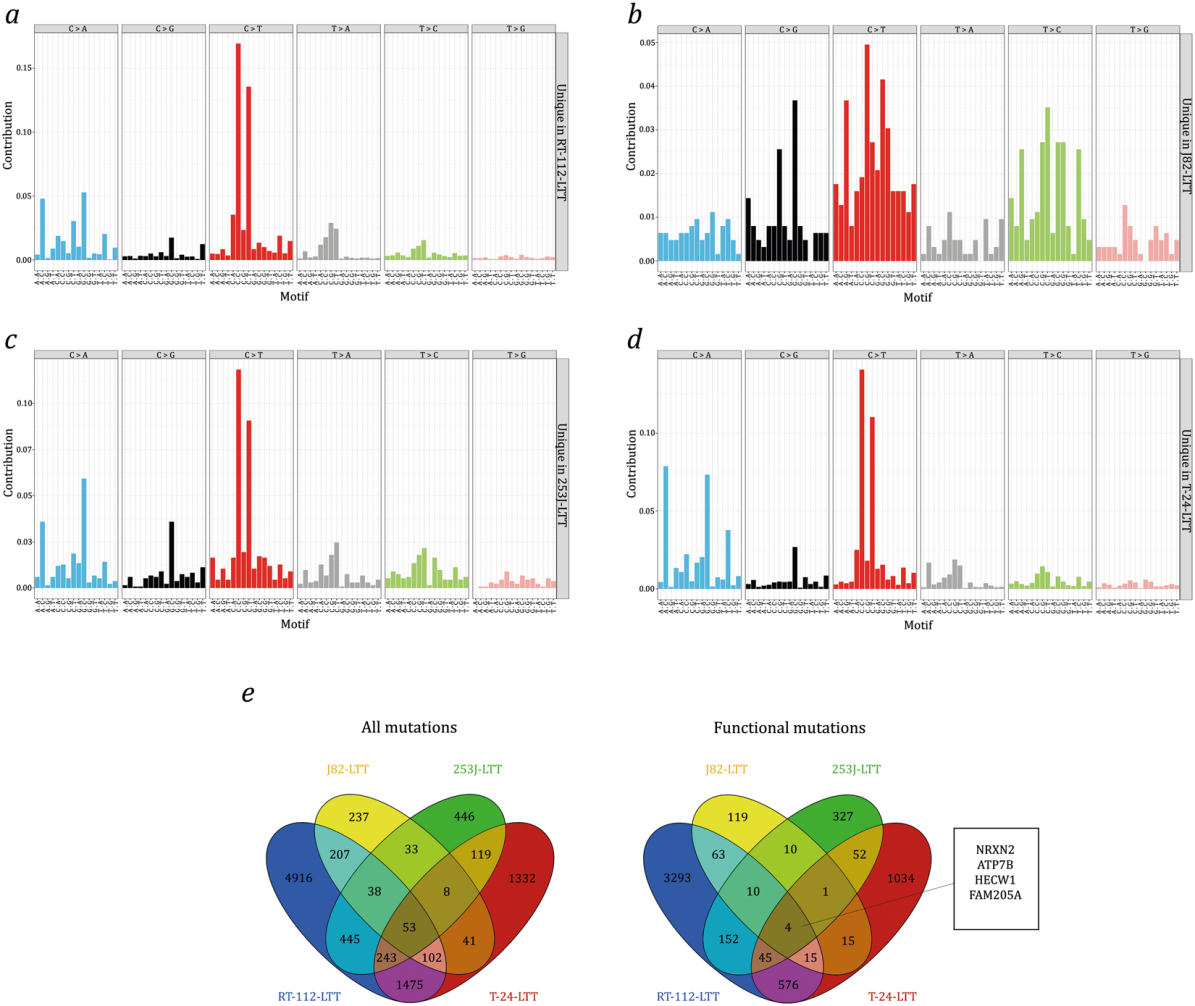
Mutation profiles of LTTs. Mutational spectra of (**a**) RT-112-LTT, (**b**) J82-LTT, (**c**) 253J-LTT, and (**d**) T-24-LTT relative to their parental UCCs are displayed according to the 96 substitution classification defined by the substitution class and sequence context of the mutated base by Alexandrov *et al*. (2014). Note that the number of mutations in J82-LTT is too low to allow the generation of a representative profile. (**e**) Common variants found in RT-112-LTT, J82-LTT, 253J-LTT, and T-24-LTT for all variants (left Venn diagram) and exonic nonsynonymous variants (right Venn diagram).

Whole exome sequencing was performed for the four parental cell lines and the four LTTs, maintained with cisplatin for 12 months. Deviations from the hg19 consensus sequence in the parental cell lines and the LTTs were determined as described in Methods and are detailed in Tables S1–S8. Of note, no normal germ-line DNA is available for the parental cell lines and it is therefore not possible to determine which of the differences in the parental cell lines towards the hg19 consensus sequence are somatic mutations rather than germ-line variants. However, all parental cell lines contain mutations in genes significantly mutated in urothelial carcinoma (TCGA-SMG, where TCGA stands for The Cancer Genome Atlas), most of which have been documented in databases (see sheets TCGA SMG in Tables S1, S3, S5 and S7). Compared to their parental lines, the LTTs acquired various additional mutations in TCGA-SMG, which are listed in Tables S9–S12. Among the LTTs, the highest number of overall novel sequence variants (Tables S2, S4, S6 and S8) was observed in RT-112-LTT cells and the lowest number in J82-LTT. Therefore, the frequency of mutations increased in parallel with the cisplatin maintenance concentrations (Fig. 1a). The majority of mutations were single base exchanges (Table 1). In three of the four LTT sublines the most frequent single base exchanges were C > T transitions and C > A transversions, however, in J82-LTT the number of mutations (n = 865) was too small to allow firm conclusions (Fig. 1b). No strand bias was obvious (Fig. 1c). The mutation profiles in the three evaluable cell lines revealed an almost exact correspondence to the cisplatin-specific signature recently published by Boot *et al*.^13^, namely frequent C > A transversions preferentially in a ACC or GCC context, more frequent C > T transitions preferentially in CCC or CCT and less commonly in CCA or CCT contexts, with rarer transversions of C > G, predominantly in a GCC context and T > A, with a preceding C and any base following (Fig. 2a–d). Cosine similarity between the mutational profiles of RT-112-LTT, 253J-LTT, and T-24-LTT each exceeded 0.94, but ranged only from 0.46 to 0.60 between these cell lines and J82-LTT (Table 2). The cosine similarities of the mutational profiles of the former three cell lines and those of HepG2 and MCF reported by Boot *et al*. (2018) ranged between 0.90 and 0.97 (Table 2).

**Table 1:** Summary of genetic variants of RT-112-LTT, J82-LTT, 253J-LTT, and T-24-LTT cells detected by exome sequencing.

Variants	RT-112-LTT	J82-LTT	253J-LTT	T-24-LTT
Overall number of mutations	12463	864	1602	4250
Number of genes	7479	720	1385	3373
Single base exchanges	11767	638	1354	3880
Double base exchanges	377	29	61	123
Insertions	139	71	60	88
Deletions	164	131	122	108
Single base deletions	90	65	59	91
Intronic	3300	280	463	1117
Exonic	7938	467	938	2760
UTR	708	84	136	213
Synonymous	2325	135	230	684
Nonsynonymous	4831	222	561	1740
Frameshift	116	49	39	73
Nonframeshift	292	37	63	109
Stop-gain	279	7	24	105
Unclassified/unknown	4615	414	685	1536

**Table 2:** Cosine similarities between RT-112-LTT, J82-LTT, 253J-LTT, and T-24-LTT compared to MCF10A and HepG2 data from Boot, Huang *et al*. (2018).

	MCF10A	HepG2	T-24-LTT	RT-112-LTT	J82-LTT	253J-LTT
253J-LTT	0.9356	0.9429	0.9584	0.9598	0.5994	
J82-LTT	0.5160	0.4806	0.4656	0.4611		
RT-112-LTT	0.9251	0.9604	0.9635			
T-24-LTT	0.8966	0.9431				
HepG2	0.9731					
MCF10A						

In addition, 29–377 double base exchanges were observed, in the same order of frequency among the cell lines, as well as 182–303 InDels (Table 1). Interestingly, single base deletions occurred preferentially between the flanking bases G and C (56/215, 26%).

Among the mutations located within coding regions, more synonymous mutations were observed than nonsynonymous (including stop-gain) mutations (27.0–59% vs. 25% expected by chance), indicating no positive selection for functionally important mutations in general.

The mutations in the four LTTs affected between 720 and 7479 genes. Only 53 protein-coding genes were mutated in all four LTTs (Fig. 2e, Table 3), most of which were larger than the estimated average gene size of 53.6 kb^15^. Among these genes, mutations likely to have a functional impact (nonsynonymous, stop-gain, or frameshift) were found in *NRXN2*, encoding the cell adhesion protein neurexin 2, *HECW1*, encoding the eponymous ubiquitin ligase (also known as NEDL1), *FAM205A*, encoding a poorly studied transmembrane protein, and *ATP7B*, a copper transporting ATPase.

**Table 3:** List of genes mutated in all four LTT cell lines.

Gene	Gene Size [kB]	Protein Size [aa]	Molecular mass [Da]	No. of LTT lines with functional mutations
*CACNA1C*	727.2	2221	248977	2
*AGAP1*	637.7	857	94470	2
*HECW1*	453.4	1606	179554	3
*HYDIN*	428.6	5121	575892	3
*IQSEC*.*1*	386.0	963	108314	0
*FAM13A*	385.4	1023	116932	0
*ACSS3*	324.8	686	74778	2
*TTN*	304.8	34350	3816030	3
*PLEKHA7*	237.1	1121	127135	1
*ADAMTS2*	234.6	1211	134755	1
*FREM2*	200.1	3169	351157	3
*PRKDC*	187.1	4128	469089	0
*COL13A1*	162.4	717	69950	1
*SEL1L2*	147.3	688	77964	2
*CFAP46*	134.8	2715	303500	1
*MUC16*	132.5	14507	1519175	2
*MAP3K4*	125.7	1608	181685	1
*TDRD9*	124.2	1382	155683	2
*ATP7B*	121.0	1465	157263	4
*LTBP2*	120.4	1821	195052	2
*NRXN2*	117.0	666	70927	4
*WDR17*	117.0	1322	147703	2
*DNAH2*	116.4	4427	507698	1
*HSPG2*	115.1	4391	468830	3
*NUP210*	104.1	1887	205111	1
*SI*	99.6	1827	209453	2
*BAHCC1*	70.9	2608	276932	0
*ZAN*	64.2	2812	305663	0
*LAMA5*	59.4	3695	399737	1
*USP36*	54.1	1121	122652	3
*NBPF1*	51.3	1214	139343	0
*ITGAL*	50.5	1170	128770	0
*KCNJ12*	43.7	433	49001	0
*KMT2D*	42.9	5537	593389	2
*SMTN*	40.7	917	99059	2
*INTS1*	34.1	2190	244297	1
*PLEKHA4*	31.5	779	85401	0
*MYH6*	26.3	1939	223735	1
*NUMBL*	25.8	609	64891	1
*HDAC6*	23.6	1215	131419	3
*FLG*	23.1	4061	435170	1
*OBSL1*	22.5	1896	206947	2
*C3orf38*	19.0	329	37541	0
*PLA2G4F*	17.6	849	95082	2
*EPS8L3*	13.9	593	66861	1
*SIK1*	12.6	783	84902	2
*FGF23*	11.5	251	27954	1
*FAM205A*	6.5	1335	148096	4
*PRB4*	3.4	310	31326	0
*PCDHB8*	2.7	801	87639	0
*SPRR3*	2.1	169	18154	0
*KRTAP5-2*	1.1	177	16271	1
*MIR1268A*	0.1	none	none	0

**Figure 3:**
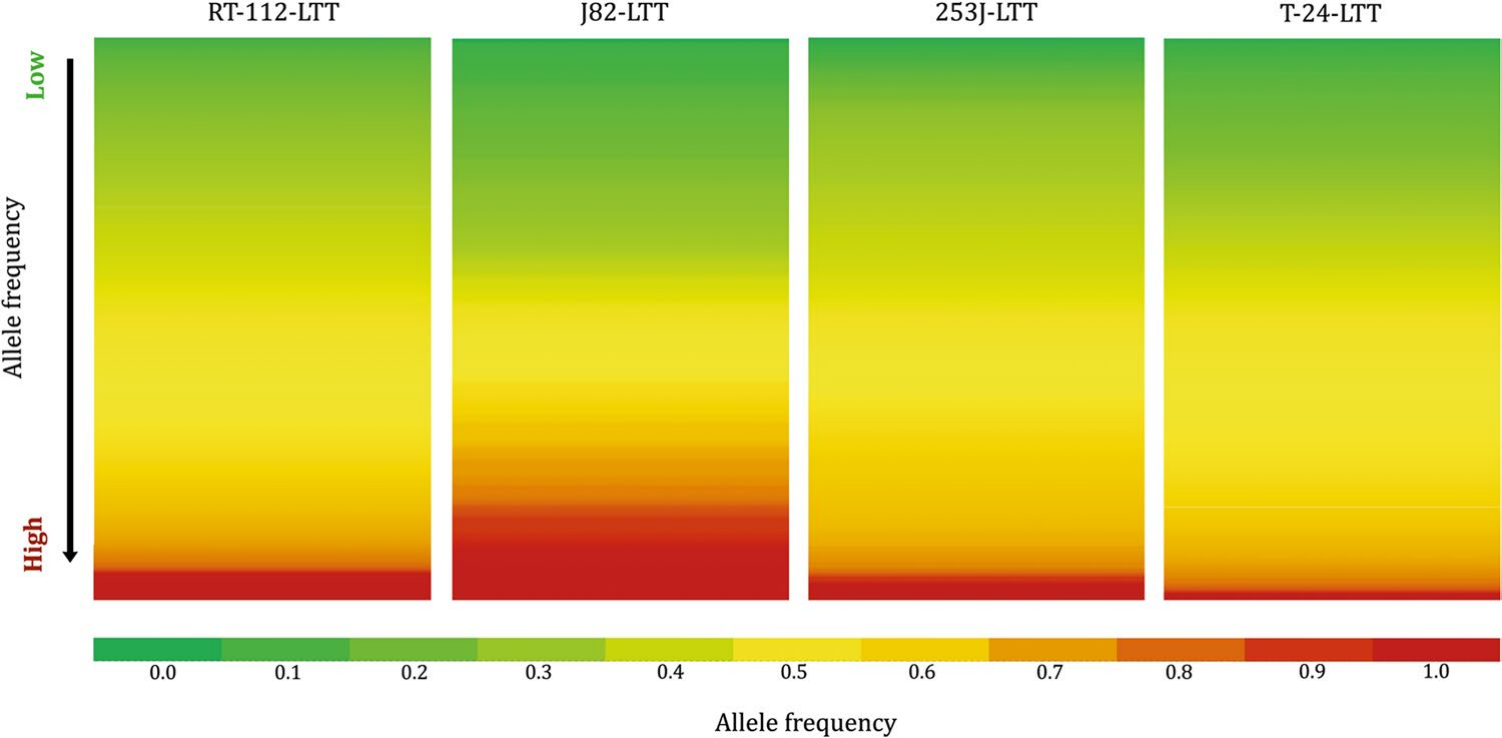
Allele frequency of mutations found in LTTs. Heat map indicating allele frequencies in four LTT sublines from low (green) to high (red).

The variant allele frequency of the new variants extended continuously to 1 in all LTTs (Fig. 3). In fact, a large number of new mutations were present at variant allele frequency close to 1 in all LTTs, indicating that they were homozygous and clonal. Moreover, many mutations with an allele frequency above 0.5 could be heterozygous and thus be present throughout the entire population. Conversely, the broad range of variant allele frequencies suggests that some mutations were only present in a subset of the cell population. This distribution is best explained by the assumption that in each LTT line some mutations occurred in a cell clone expanding during the initial selection process for cisplatin resistance, but others were acquired gradually during further expansion.

**Figure 4:**
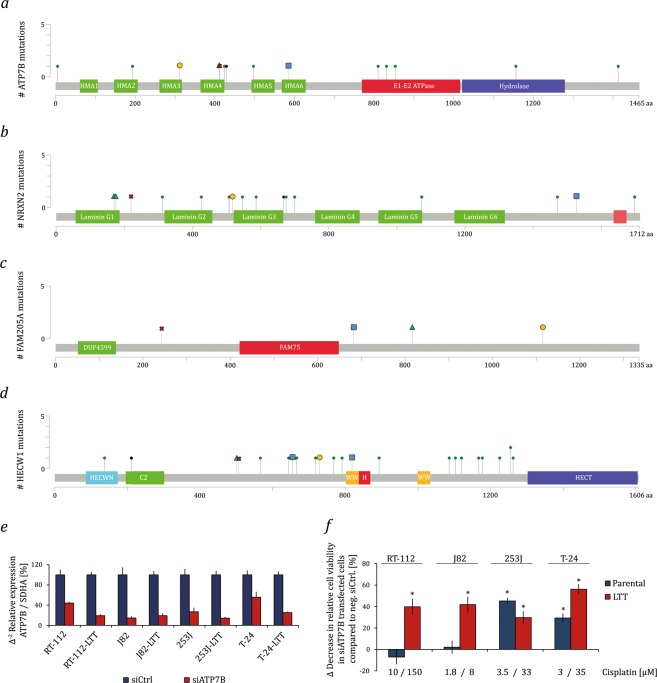
Protein domain structure of genes commonly mutated in LTTs. Overview of the domain structures of (**a**) ATP7B, (**b**) NRXN2 (Neurexin-2), (**c**) FAM205A, and (**d**) HECW1 (NEDL1). Mutations found in the TCGA data set as annotated from cBioportal and mutations found in LTTs were included. All mutations in the LTTs are missense mutations except for one FAM205A mutation in RT-112 (Table S14). (**e**) ATP7B mRNA expression was measured by qRT-PCR in parental UCCs and LTTs after treatment with siRNA against ATP7B or a non-targeting control. SDHA mRNA was used as a reference. (**f**) Relative cell viability after 48 h cisplatin treatment as measured by CellTiterGlo assay in parental UCCs and LTTs transfected with siRNA targeting ATP7B or a non-targeting negative control siRNA. Values represent the mean ± SD of biological quadruplicates, *p < 0.05 compared to non-targeting control. HECT: Homologous to the E6-AP Carboxyl Terminus; HECWN: HECTW1 N-terminal domain; H: Helical bundle; C2: C2 domain; WW: WW domain; HMA: Heavy metal-associated domain.

Most interestingly, all LTTs contained missense mutations in *ATP7B*, and RT-112-LTT additionally contained mutations in *ATP7A* (Tables S13 and S14). Both genes encode copper transporters located in the plasma membrane which support also cisplatin extrusion^16^. The missense mutations in *ATP7B* are distributed throughout the protein at allelic frequencies of 0.45–1 (Fig. 4a). Similar to the mutations found in the TCGA data, mutations in *ATP7B* were mostly observed in the heavy metal-associated domains (HMA, Fig. 4a, Table S14). These domains are required for binding of copper and platinum ions and subsequent activation of ATP-dependent metal ion export^16^. The functions of two further genes mutated in all 4 LTTs, namely *NRXN2* (allelic frequencies 0.31–0.42) and *FAM205A* (allelic frequencies 0.28–0.85), are entirely unexplored in the context of cancer and chemoresistance. *NRXN2* encodes Neurexin-2, a plasma membrane and adhesion protein predominantly expressed in the brain. Most of the mutations found in *NRXN2* were observed in the first three Laminin domains (Fig. 4b, Table S14). The FAM205A protein is poorly characterized to date; it is predominantly expressed in testis and ovary. In the LTTs, mutations found in *FAM205* were distributed throughout the gene (Fig. 4c, Table S14). A fourth gene, *HECW1*, is a more plausible candidate for being functionally involved in chemoresistance. The gene encodes the E3 ubiquitin ligase NEDL1; among its substrates is the WNT/β-catenin pathway signal transducer DVL1. The *HECW1* mutations in the LTTs were found at allelic frequencies of 0.16–0.59 and, according to the TCGA data, in a commonly mutated region (Fig. 4d, Table S14).

To exemplarily explore the impact of the mutations in *ATP7B* on cisplatin sensitivity, we used an siRNA mediated knockdown approach. Parental and LTT cell lines were transfected with siRNA targeting ATP7B or a non-targeting control siRNA. Following successful knockdown (Fig. 4e) cells were treated with cisplatin at IC_50_ concentrations (parental cell lines) or maintenance concentrations (LTTs). Knockdown of ATP7B enhanced the effect of cisplatin in all LTTs, but interestingly also in 253J and T24 parental cells (Fig. 4f).

The number of genes affected by sequence changes in the LTTs was high, even considering only potentially functional mutations (i.e. nonsynonymous, frameshift and stop-gain). It is therefore not clear which mutations functionally contribute to cisplatin resistance. Accordingly, an analysis using the DAVID functional annotation tool^17^ revealed predominantly structural rather than functional characteristics to be enriched among the mutated genes. In the three cell lines with a higher number of mutations, the top significant keywords were polymorphism, alternative splicing, phosphoprotein and coiled-coil-domain. Strictly functional attributes were only significant in T-24-LTTs, where genes encoding ECM (extracellular matrix) and cell adhesion molecules were overrepresented among the mutated genes. However, these attributes may rather reflect that larger genes are more likely to be hit. We therefore searched specifically for mutations in genes previously implicated in mechanisms of cisplatin resistance (abbreviated CRG here) as well as for TCGA-SMG. Among the CRG, a *KEAP1* mutation previously described in RT-112-LTT^8^ is very likely to contribute to NRF2 activation in this cell line, as KEAP1 negatively regulates NRF2. A mutation in *BACH1*, albeit relatively conservative (A678G) might influence the same pathway. Mutations in three genes, *CHUK* (G289R), *NFKB1* (nonframeshift substitution) and *SIRT6* (G22A) might alter NFκB survival signaling. A different, presumably heterozygous mutation of *CHUK* (A42E) was also detected in 253J-LTT. Three LTT lines acquired mutations in *HDAC6*, a histone deacetylase involved in cell stress responses. Most interestingly, all LTTs contained missense mutations in *ATP7B*, and RT-112-LTT additionally contained mutations in *ATP7A*, which encode copper transporters thought to support cisplatin extrusion (Table S13).

Among the TCGA-SMG, RT-112-LTT gained likely functionally relevant mutations in *TP53* (R198H), *FOXA1* (A83T in the forkhead N-domain) at high allele frequency, *ERCC2* (Y16N in the helicase domain), *ARID1A* (G406R and R1566K) and *STAG2* (G667T) at presumable heterozygote frequency. Interestingly, 253J-LTT acquired two mutations in *ARID1A* (R1566K and P1567A) as well, albeit at moderate allelic frequency. All TCGA-SMG mutations in T-24-LTT occurred at relatively low variant allele frequencies and none were detected in J82-LTT (Table S13).

### Chromosomal changes

**Figure 5:**
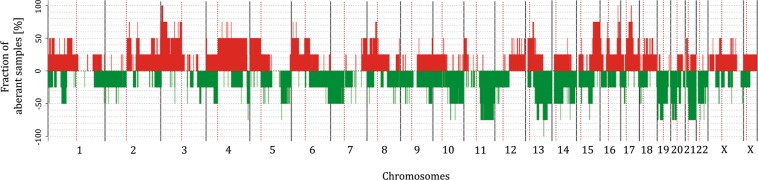
Common chromosomal alterations across all LTT sublines as detected by aCGH. 100% denotes change in all cell lines.

**Figure 6:**
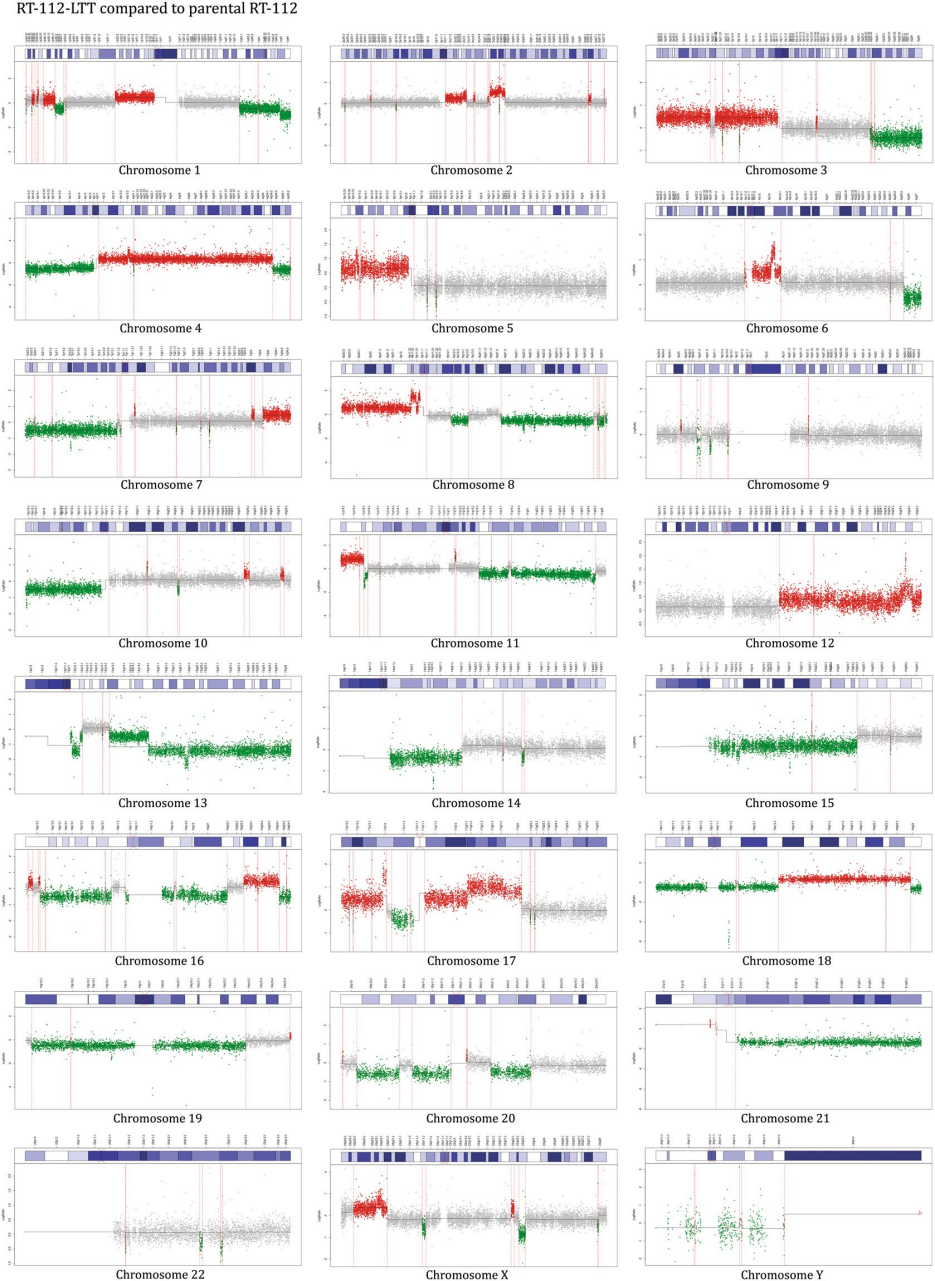
Chromosomal alterations in RT-112-LTT by aCGH. Chromosomal alterations of RT-112-LTT hybridized against its parental cell line as detected for each chromosome by aCGH.

**Figure 7:**
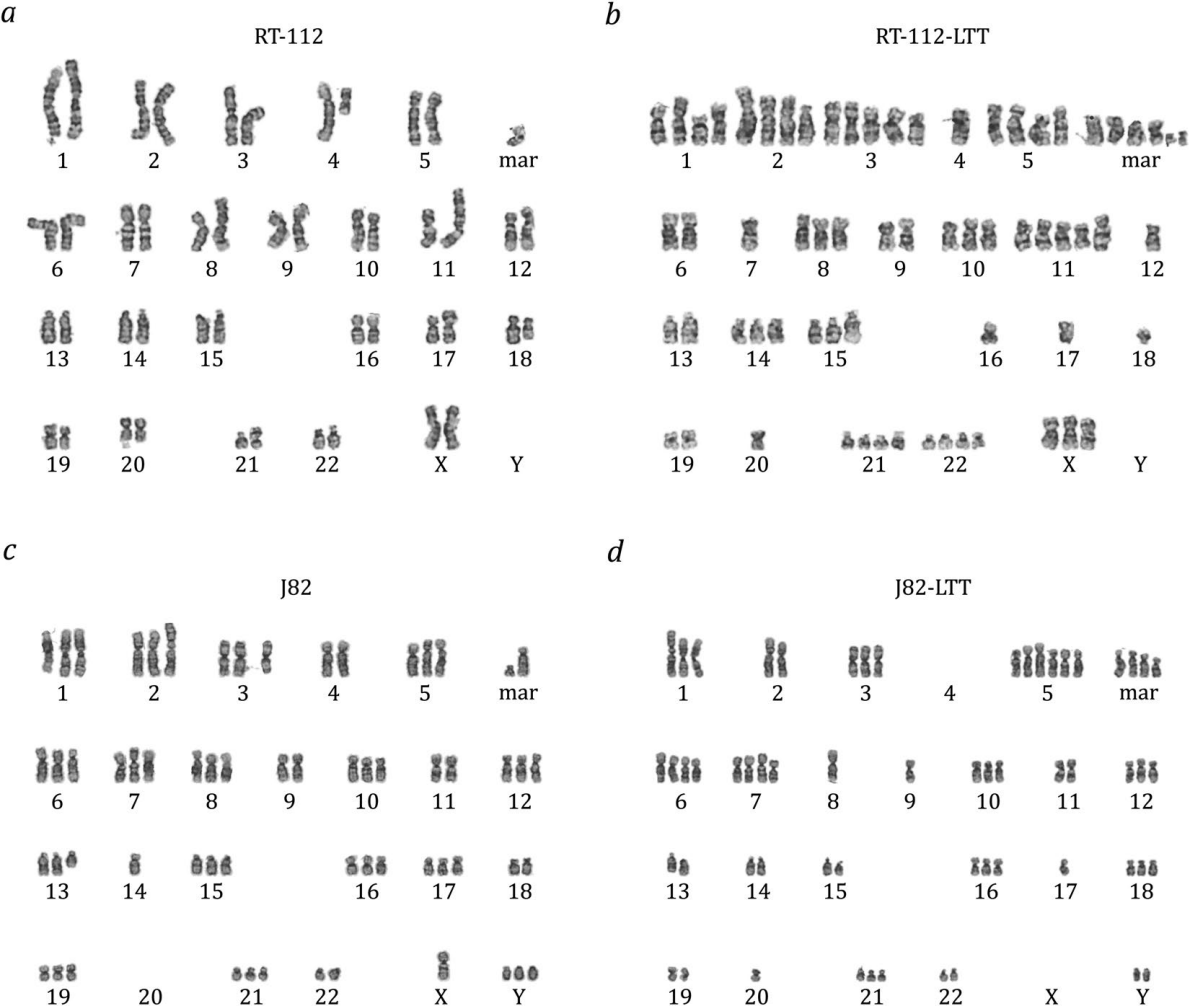
Karyotype changes in LTTs. Karyotype of (**a**) RT-112, (**b**) RT-112-LTT, (**c**) J82, and (**d**) J82-LTT as revealed by GTG-banding analysis.

To detect karyotype changes in the LTT lines, array-CGH was performed for all four cell line pairs. Since all four cell lines are aneuploid, rather than comparing each cell line against a reference genome and then calculating the differences between parental and LTT variant, DNA from each parental cell line was hybridized against that of its LTT variant (Figs 5, 6 and S1–S3, Table S15). The validity of this approach was ensured by hybridizing one pair of cell lines to a reference genome mixture, which yielded comparable results, but was less sensitive for differences, as expected (Fig. S4, Table S16). All four LTTs contained multiple changes compared to their parental cell lines which affected most or all chromosomes (Fig. 5). The changes are detailed for RT-112 in Fig. 6, a normally karyotypically relatively stable cell line with a near diploid chromosome number as revealed by conventional chromosome banding analysis (46,XX,add(1)(p34),del(3)(p12p21),i(4)(p10),i(8)(q10),add(11)(p15),add(17)(p12),del(18)(q21),i(21)(q10) in our variant (cf.^18^). In contrast, RT-112-LTT cells had a significantly higher number of chromosomes compared to RT-112 parental cells (64.4 ± 1.9 vs. 45.6 ± 1.9, *p* < 0.0001) including several new markers (Fig. 7a,b). The other three parental cell lines are highly aneuploid, making karyotyping by chromosome banding analysis more difficult. However, mean chromosome numbers were also significantly increased in T-24-LTT vs. T-24 (Fig. S5a,b; 68.2 ± 2.4 vs. 79.9 ± 4.0, *p* < 0.0001) and decreased on average in J82-LTT vs. J82 (Fig. 7c,d; 73.0 ± 12.2 vs. 65.2 ± 11.9, *p* = 0.064), likewise with new markers (Fig. 7c,d). A slight and nonsignificant increase in chromosome number was seen in 253J-LTT over 253J (Fig. S5c,d; 54.1 ± 1.2 vs. 57.4 ± 5.0, *p* = 0.064).

In the aCGH analyses, chromosomal instability presented as multiple segmental chromosomal gains and losses as well as focal deletions and amplifications, as illustrated exemplarily for RT-112-LTT in Fig. 6 (see Figs S1–3 for the other LTTs). Interestingly, whereas J82-LTT had the lowest number of point mutations, copy number changes were observed for each chromosome in this cell line, as in RT-112-LTT and T-24-LTT. The lowest change in mean chromosome numbers was instead observed in 253J-LTT, where several chromosomes appeared to be unaltered, although other chromosomes contained typical segmental gains or losses.

**Figure 8:**
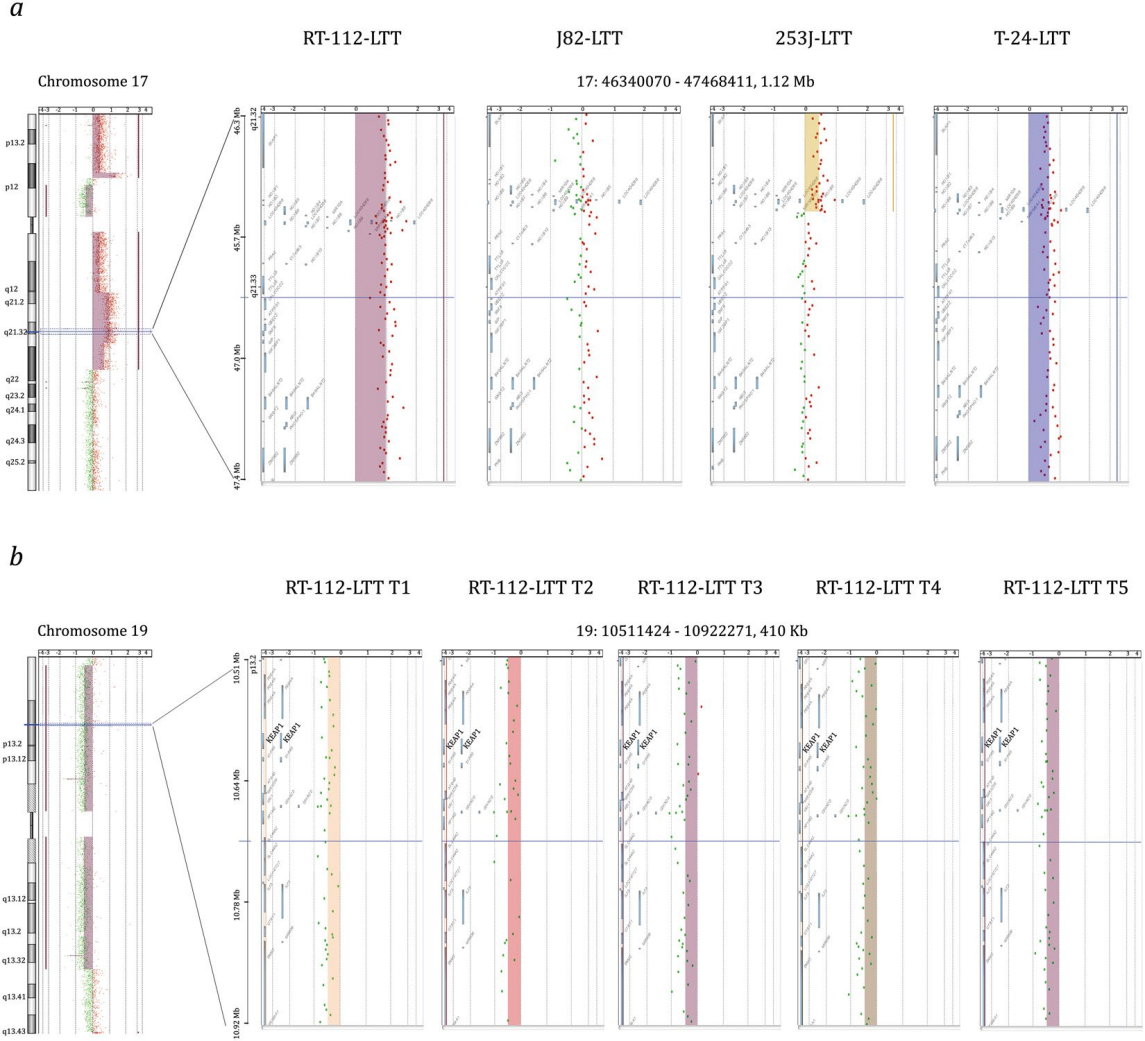
Exemplary chromosomal changes in LTTs. (**a**) The region 17q21.32 around the *HOXB* gene cluster was gained in all four LTTs as detected by aCGH. (**b**) The region 19p13.33 containing the *KEAP1* gene was lost in RT-112-LTT at five different time points (T1–T5) according to aCGH.

Only few chromosomal gains or losses were shared between the LTT cell lines (Table S15), suggesting that most gains or losses do not contribute to cisplatin resistance. All four UCC-LTTs had gains in chromosomal regions 3p26.2-p25.3, 16q23.1 and 17q21.32, as well as losses in 13q31.4 (Figs 5, S1–3 and 8a). At least three of the four UCC-LTTs had gains and losses in chromosomal regions including the genes *SLC6A11*, *SLC6A1* (within gain 3p25.3-p24.3), *IKKB*, *POLB*, *SLC20A2*, *DKK4* (gain 8p11.23-p11.21), *MRE11A*, *MRE11B* (loss 11q14.2-q21), *BCL2A1* (gain 15q24.3-q25.3), *BLM* (gain 15q26.1-q26.3), *TOP2A*, *ERBB2*, *KRT14*, *STAT3*, *BECN1*, *BRCA1*, *und WNT3* (gain 17q12q-21.32). Many of these genes have functions relevant for cisplatin resistance, such as cell survival, DNA repair and WNT signaling^9^. The *KEAP1* copy number in the 19p13.3-q13.33 region appeared diminished in all cell lines, including RT-112 (Fig. 8b).

In order to determine when the copy-number changes had arisen, aCGH profiles were generated for two LTT lines from 4 different time points, starting with the first available expanded passage. The majority of the changes seen in the late passage LTTs were already established at the first available time point, but some changes accrued during further passages (Figs 8b, S6a–d and S7a–d, Tables S17 and S18).

## Discussion

In four independent urothelial carcinoma (UC) cell lines selected for resistance to high concentrations of cisplatin, we observed a large number of mutations with distinctive characteristics by whole exome sequencing and pronounced chromosomal instability by aCGH. Since all parental cell lines can be safely considered as optimally evolved for long-term propagation in culture, mutations and chromosomal changes observed in the resistant LTTs are expected to reflect the genotoxic impact of cisplatin and potentially positive selection for resistance to the drug, but not selection for other properties like faster growth. In fact, the LTTs grow more slowly than their respective parental cells^5^.

Two of our questions were to which extent cisplatin induced point mutations in the cell lines during selection and whether these mutations had a distinct profile. The number of point mutations, ranging from 865 to 12,500, largely paralleled the tolerated drug doses. By comparison, the parental cell lines harbor between 34,400 and 36,800 variants compared to the reference genome, meaning that between 2.3% and 34.1% mutations were additionally induced. These are minimum percentages, as a fraction of the variants in the parental cells will be germ-line variants and as we did not consider rare variants (i.e. below 10% of all reads) in the LTTs. Thus, treatment with cisplatin induced a large number of mutations.

Currently, five cancer signatures have been identified as prevalent in bladder cancer, more precisely, signatures 1, 2, 5, 10, and 13^19,20^. The profiles of new mutations in the three UC LTTs with sufficient numbers of mutations fitted exactly with that defined by Boot *et al*. for hepatoma HepG2 and breast cancer MCF7A cells^13^. Thus, across three different human tumor entities, cisplatin appears to elicit the same mutation profile. Moreover, the mutation profile seen by Boot *et al*.^13^ and in our analysis is highly similar to a new mutational signature derived by Liu *et al*.^21^ from a comparison of UC tissue samples before and after treatment with cisplatin-based chemotherapy, which in turn bears strong similarities to that observed in another study on post-chemotherapy metastatic urothelial carcinomas^13,21,22^. Liu *et al*. proposed that this signature is likely caused by cisplatin^21^. Our results strongly support this contention. The newly defined cisplatin mutational signature resembles the cisplatin mutation profile previously described in *C*. *elegans*, *S*. *cerevisiae*, and DT-40 chicken cells^23–25^ in its predominance of C > A and C > T changes, but the profiles differ in their base contexts, suggesting differences in cisplatin mutagenicity between species. Of note, in addition to the distinctive three-base contexts, we observed further characteristics of cisplatin mutagenesis in the LTTs, such as double-base replacements and single base deletions. On a note of caution, the cisplatin mutational signature cannot be identified in J82-LTT. While this is plausibly due to the low number of mutations in this cell line, we cannot formally exclude that it presents a different mutational signature.

Whereas the spectrum of cisplatin-induced point mutations observed in our study in urothelial carcinoma cell lines is very similar to that observed in UC tissues following neoadjuvant chemotherapy *in vivo*, the relative number of mutations differs strongly, as Liu *et al*. observed only a low percentage of new mutations (<1%)^21^. Plausible explanations for this difference are (i) the higher drug concentrations used on the cell lines, as supported by the positive association between cisplatin maintenance concentration and number of mutations, and (ii) the longer treatment duration in the cell lines, as supported by the large fraction of subclonal mutations in an overall clonal environment, which suggests accrual over time. Further potential factors accounting for the difference could be the use of drug combinations in patient treatment and more pronounced tumor heterogeneity *in vivo*. Notably, unlike Liu *et al*. in patient tissues, we did not observe APOBEC-related mutational signatures for the new variants in the LTTs, even though UC cell lines express APOBEC3B^26,27^.

The same factors may also account for the discrepancy between the high chromosome instability detected in the LTTs and the low number of copy number alterations (CNA) reported by Liu *et al*. in bladder cancer tissues following chemotherapy^21^. In addition, detection of CNAs by aCGH in the pure tumor cell population in cell lines is likely to be more sensitive and robust than inference of CNA from exome sequencing of cancer tissues with a variable stromal content. Cisplatin is known to induce chromosomal aberrations, presumably mostly as a consequence of interstrand crosslinks. Accordingly, cisplatin sensitivity is related to the cellular capacity for interstrand crosslink and homologous recombination repair. In the bladder cancer cell lines used, interstrand crosslink repair is functional^28–30^. In the LTTs, several new variants in DNA repair genes were detected, but all occurred at relatively low allelic frequency and almost all were silent or conservative changes. Most UC cell lines, however, are deficient in DNA damage checkpoint signaling due to inactivation of p53 and RB1 function, a defining property of almost all urothelial cancers^31^, including those investigated by Liu *et al*.^21^. Therefore, the different extent of CNAs is unlikely to result from differences in DNA repair and checkpoint signaling between tumor tissues and cell lines. As for the number of mutations, the intensity and duration of cisplatin treatment may represent the decisive factor. The lower amount of chromosomal aberrations in 253J-LTT cells is in line with this idea. Our results therefore clearly indicate the potential of cisplatin to induce severe chromosomal aberrations, while the comparison to the tissue data suggests that their extent may depend on drug concentration and treatment duration. These dependencies deserve further investigation to better understand the clastogenic effects of cisplatin and their impact in patients.

Both the results from WES and aCGH argue that the LTT cell lines were generated by the initial selection for a surviving clone with multiple new mutations and chromosomal alterations followed by gradual acquisition of further mutations and - albeit to a limited degree - of copy number changes. From the WES data, this conclusion is supported by the relatively high fractions of homozygous (or near homozygous) new mutations in each cell line (Figs 1 and 3) accompanied by a substantial fraction of new mutations at lower frequency. More direct evidence for the temporal sequence is provided by the aCGH comparison of LTTs over time showing that most of the changes observed after 12 months of culture were present in the first experimentally accessible cell population after initial selection. Of note, this sequence of events can be straightforwardly accommodated with the cell treatment modus, as following the initial selection period with increasing concentrations of cisplatin, LTTs were treated after each passage with cisplatin concentrations much higher than the respective IC_50_s of their parental cells, but below their acquired IC_50_. These concentrations still induce DNA damage as evidenced by accumulation of cells in S-phase and growth arrest, from which the LTTs - unlike parental cells - recover within a few days^7^. Thus, during the initial selection period, more DNA damage is incurred, whereas during further propagation, cells sustain DNA damage only to an extent with which they can cope. Accordingly, any mutagenic effects of cisplatin will also be less dramatic.

Notably, clonal selection in the LTTs may constitute another factor explaining the higher extent of CNAs in the cell lines compared to tumor tissues. In the cell model, a single clone apparently was selected from a relatively homogeneous cell population. Cancers *in vivo* are more likely to be genomically heterogeneous at the start of treatment and populations emerging during treatment are oligoclonal^21,22^. In such tumors, point mutations are more easily detected than copy number changes by current deep sequencing techniques.

Because of the high number of mutations and copy number changes in the LTTs, our last question, namely whether genomic changes may underlie the observed mechanisms of resistance in these cells, is difficult to answer. In addition to the previously reported *KEAP1* mutation promoting NRF2 activation in RT-112-LTT^8^, we detected mutations or copy number changes in genes related to NF-κB-signaling, DNA repair and cell survival regulation, among others. In addition, several point mutations affected large chromatin regulators like *ARID1A*, which are known to be relevant in UC in general and are important for transcriptional regulation of cell survival. Whether these genomic alterations are functionally important needs to be ascertained individually.

Cases in point are the frequent missense mutations observed in the *ATP7A* and *ATP7B* metal ion transporters. Both are capable of exporting platinum compounds and are established as contributing to cisplatin resistance^16^. Specifically, mutations in *ATP7B* have been observed in patients with metastatic bladder cancer and may be predictive for the response to cisplatin-based chemotherapy^32^. In the LTTs, expression of *ATP7A* and *ATP7B* is at most moderately changed^7^, but the present findings raise the possibility that missense mutations, especially in *ATP7B*, might contribute to cisplatin resistance. These mutations are located within the HMA (heavy metal-associated) domains in the N-terminal metal-binding region where site-directed mutations have been demonstrated to influence cisplatin binding and ATPase activation^16^. Interestingly, some mutations described in the TCGA data set are also located in HMA domains. Our data thus support the assumption that mutations in *ATP7B* may be a significant mechanism in the development of cisplatin resistance in UC. As a further support of their functional importance this idea, we observed that siRNA-mediated knockdown of ATP7B sensitized all four LTTs to cisplatin. The precise effect of each missense mutation on cisplatin binding and extrusion would however need to be tested in appropriate models^16^.

In addition, the functional significance of mutations in *HECW1* deserves further investigation^33^. Its gene product, NEDL1, is best characterized as an ubiquitin ligase for the WNT/β-catenin pathway signal transducer DVL1 and its loss of function could therefore increase the activity of the pathway. Increased activity of this pathway has previously been implicated in cisplatin resistance^34^ and, interestingly, we have observed increased expression of some of pathway targets in the LTTs^5^. NEDL1 is moreover capable of inducing apoptosis in a p53-dependent manner. The UC cell lines used in our study are all p53-mutant, but the influence of NEDL1 on apoptosis in UC cell lines should likewise be investigated in future work.

In conclusion, our investigation demonstrates that the emergence of cisplatin-resistant UC cell lines in a continuous treatment model is accompanied by massive genomic changes, both point mutations and chromosomal alterations. These findings underline the potential of cisplatin to cause genomic changes in cancers and patients. Our data confirm a mutational signature proposed by others^13,21^, which might be used to detect the impact of cisplatin with respect to point mutations *in vivo*. For several reasons, including polyclonality and tumor heterogeneity, copy number changes are more difficult to detect *in vivo*. However, if detectable, segmental gains and losses as well as focal alterations of the sort seen in the LTTs could be indicators of cisplatin clastogenic activity in tumor and other tissues.

## Material and Methods

### Cell culture and treatment

The human UC cell lines (UCCs) RT-112 and T-24 were obtained from the DSMZ (ACC 418, ACC 376), whereas 253J and J82 cell lines were kindly provided by J. Fogh (New York, USA). Parental UCCs and their long-term cisplatin-treated sublines were grown in DMEM GlutaMAX-I (Gibco, Darmstadt, Germany) containing 10% heat-inactivated fetal bovine serum. Long-term cisplatin-treated sublines (LTTs) were established by continuously escalating cisplatin (Accord Healthcare, Muenchen, Germany) dosages over several months until stable resistant cell lines could be maintained at doses of 50 μM (RT-112-LTT), 3.3 μM (J82-LTT), 6.6 μM (253J-LTT), and 23 μM (T-24-LTT) as described previously^5^.

### SiRNA-mediated knockdown, validation and measurements of cell viability and apoptosis

The siRNA-mediated knockdown of ATP7B in UCCs was performed by transfecting 10 nmol/L siATP7B or a non-targeting control (#L-019281-00-0005 and #D-001810–10-05, both Dharmacon, Lafayette, CO, USA) using Lipofectamine RNAiMAX Reagent (Thermo Fisher, Waltham, MA, USA) according to the manufacturer’s protocol. RNA isolation, cDNA synthesis, and quantitative real-time PCR were performed as previously described on the Lightcycler 96 system (Roche, Basel, Switzerland)^5^ using primers amplifying ATP7B (Fwd. TACCCATTGCAGCAGGTGTC; Rev. ACTTGAGCTGCAGGGATGAG) or the housekeeping gene SDHA (Fwd. GCCAGGACCTAGAGTTTGTTCA; Rev. CTTTCGCCTTGACTGTTAATGA) for normalisation. Relative expression was calculated by the 2^−ΔΔCt^ method Relative cell viability was measured in quadruplicates by the CellTiter-Glo assay (Promega, Fitchburg, WI, USA).

### Karyotyping

For chromosome preparation the cells were grown to near confluency, and incubated with 10 μg/ml colcemid (Biochrom, Berlin, Germany) at 37 °C for 0.5–2 hours. Cells were harvested by using 0.05% trypsin/ 0.02% EDTA (ethylene diamine tetraacetic acid) in phosphate-buffered saline (PBS) (Cytogen, Wetzlar, Germany), treated by hypotonic solution (0.56% KCl w/v) for 30 min at room temperature, and fixed in ice cold methanol/acetic acid solution (3/1, v/v). The fixed cells were dropped onto ice cold wet slides, and air dried. Giemsa banding using Trypsin and Giemsa (GTG-banding) was performed according to standard protocols. For chromosome banding analysis a digital karyotyping system (Metasystems, Altlussheim, Germany) was used. A total of 20 metaphases of each cell line were analyzed. The karyotype was designated according to the International System for Human Cytogenetic Nomenclature^35^.

### DNA extraction and arrayCGH analysis

DNA was extracted by a Blood & Cell Culture DNA Midi Kit (Qiagen, Hilden, Germany) according to the manufacturer’s protocol. Comparative genomic hybridization (CGH) was performed using an array of 60mer DNA-oligonucleotide probes with a median overall probe spacing of 13 kb. Array CGH was done essentially according to the protocol of the manufacturer (Agilent, Santa Clara, California) using 1.0 μg of each cyanine 3-dUTP (Cy3) labelled tumor DNA and cyanine 5-dUTP labeled (Cy5) reference DNA tumor DNA (Agilent, Santa Clara, California).

Microarrays were scanned using an Agilent Technologies Scanner G2505C. Agilent’s Feature Extraction Software version 11.5.1.1 was used to extract log-ratios from the scanned images. The bioinformatic analysis was carried out with a custom-built script for the R Statistical Environment^36^, using freely available R packages from Bioconductor (bioconductor.org) and CRAN (cran.r-project.org). The R-script operated as a pipeline implementing the following steps: 1) Conversion of the reference/tumor log_10_-ratios as returned by the feature extraction software to tumor/reference log_2_-ratios. 2) Removal of improper oligo-nucleotides, flagged by the feature extraction software as having a too inhomogeneous distribution of pixel intensities throughout the spot representing the oligo (flags gIsFeatNonUnifOL = 1 or rIsFeatNonUnifOL = 1), or as being oversaturated (flags gIsSaturated = 1 or rIsSaturated = 1) or undersaturated or otherwise nonsensical (log-ratio set to zero). 3) Breakpoint detection using three different R packages: DNAcopy^37^, GLAD (algorithm: “lawsglad”)^38^, and HaarSeg^39,40^. For GLAD and HaarSeg the recommended default parameters were used; for DNAcopy, the parameter “UNDO.SD” was calculated as 0.25 / derivative log ratio spread (data), adjusting the approximate minimum log-ratio difference between adjacent segments to our own log-ratio cutoff (see point 4). 4) Assignment of an aberration status (gain, loss, normal) to the regions defined by the breakpoints based on the following criteria: To be called aberrant, a region had to span at least five oligos with an average log_2_-ratio >0.25 for duplications and <−0.25 for deletions, and it had to be supported by at least two of the three breakpoint methods used. 5) Creation of a detailed report listing the calls, their positions, sizes, log-ratios, and affected genes. For detecting recurrent aberrations, a second R-script was used to combine the results of multiple array analyses into one, reporting the relative number of samples sharing the same aberrations in textual form and as graphics (penetrance plots). For further visual inspection Agilent Workbench Software (Version 7.0) was used.

### Next generation exome sequencing and data analysis

The exome NGS (next generation sequencing) library was generated from high quality genomic DNA from RT-112, J82, 253J, and T-24 parental and LTT cell lines with the Ion AmpliSeq™ Exome RDY Kit (Life Technologies, Germany). Amplification and adapter ligation were performed according to the manufacturer’s protocol (MAN0010084 Rev.C, Life Technologies). The library was barcoded during the ligation reaction using the Ion Xpress™ Barcode Adapter Kit (Life Technologies). Library concentration was determined by Qubit measurement (Life Technologies) and library fragment length was analyzed using the 2100 Bioanalyzer (Agilent Technologies, Germany). The library was diluted to a final concentration of 8 pM. Emulsion-PCR and enrichment were performed on the Ion OneTouch™ 2 System (Life Technologies) using the Ion PI™ Hi-Q™ OT2 200 Kit following the manufacturer’s protocol (MAN0010857 Rev.B, Life Technologies). Sequencing was performed on the Ion Proton^TM^ System (Life Technologies) using Ion PI™ v2 chip with the Ion PI™ Hi-Q™ Sequencing 200 Kit according to the manufacturer’s protocol (MAN0010947 Rev.B, Life Technologies). Amplicon sequences were aligned to the human reference genome GRCh37(hg19) in the target region of the AmpliSeq™ exome using the Torrent Suite^TM^ software 5.2.0 with the implemented TMAP algorithm (Life Technologies). Subsequently, variants were detected using the Torrent Variant Caller Plugin v5.2.0.34 and the predefined parameter set ‘germ line low stringency’. Detailed annotations to each detected variant were collected in the databases of the NCBI Reference Sequence Database (Ref-Seq, version from 2015/03/22), Exome Sequencing Project (ESP6500), 1000 Genomes Project (1000 g), ClinVar (2015/06/29) and dbSNP142 using the open source software tool ANNOVAR. Further information was added by the implemented prediction tool MutationTaster. The Integrative GenomicsViewer (IGV 2.4.4) was used to visualize the read alignments and to check possible errors, for example due to homopolymeric stretches. Further information on filtering parameters and according numbers of variant is given in Supplementary Table S19.

### Mutation profiles

There are six classes of base substitution if all substitutions are referred to by the pyrimidine of the mutated base pair (C > A, C > G, C > T, T > A, T > C, and T > G). Each of these classes can be subdivided into 16 groups by sequence context if the bases immediately 5′ and 3′ to each mutated base are considered^41,42^. We calculated the relative contributions of these 96 mutated trinucleotides for each sample using the SomaticSignatures R package version 2.14.0^43^. We closer examined the base substitutions caused by cisplatin long-term treatment in each cell line by removing mutations identical in LTT and control and calculating the relative contributions of mutations that were exclusively present in each LTT cell line. The fraction of mutations at the 96 mutated trinucleotides are represented in bar charts. Cosine similarity among the mutational profiles of the LTT lines and to those of HepG2 and MCF10A (Table S20), which were kindly provided by Drs. Arnoud Bout and Steven Rozen, were calculated using the Cosine Similarity Calculator at scistatcalc.blogspot.com^44^.

### Use of the cBioPortal data base

ATP7B, HECW1, NRXN2, and FAM205A mutations in 404 bladder urothelial carcinoma samples (TCGA) were analyzed using the cBioPortal for Cancer Genomics^45,46^.

## Supplementary information

Supplementary materials

Suppl Table 1

Suppl Table 2

Suppl Table 3

Suppl Table 4

Suppl Table 5

Suppl Table 6

Suppl Table 7

Suppl Table 8

Suppl Table 9

Suppl Table 10

Suppl Table 11

Suppl Table 12

Suppl Table 13

Suppl Table 14

Suppl Table 15

Suppl Table 16

Suppl Table 17

Suppl Table 18

Suppl Table 19

Suppl Table 20
