# Supplementary material for: Distinctive mutational spectrum and karyotype disruption in long-term cisplatin-treated urothelial carcinoma cell lines
Source: Sci Rep. 2019 Oct 9;9:14476. doi: 10.1038/s41598-019-50891-w (PMC6785536; doi:10.1038/s41598-019-50891-w)
Supplement: Supplementary file 1 — Supplementary materials [file 41598_2019_50891_MOESM1_ESM.pdf]

Distinctive mutational spectrum and karyotype disruption in long-term cisplatin-treated urothelial carcinoma cell lines

Margaretha A. Skowron[1], Patrick Petzsch[2], Karin Hardt[3], Nicholas Wagner[1], Manfred Beier[3], Stefanie Stepanow[2], Matthias Drechsler[3], Harald Rieder[3], Karl Köhrer[2], Günter Niegisch[1], Michèle J. Hoffmann[1], Wolfgang A. Schulz[1*]

[1] Dept. of Urology, Medical Faculty, Heinrich Heine University Düsseldorf, Germany

[2] Biological and Medical Research Center (BMFZ), Heinrich Heine University Düsseldorf, Germany

[3] Institute for Human Genetics, Medical Faculty, Heinrich Heine University Düsseldorf, Germany

* Corresponding author: Wolfgang A. Schulz, Department of Urology, Medical Faculty, Heinrich Heine University Düsseldorf, Germany, Universitätsstraße 1, 40225 Düsseldorf, Germany, Tel: (+49)-211-8115845, Fax: (+49)-211-8115846, E-mail: wolfgang.schulz@uni-duesseldorf.de

Supplementary figure 1

J82-LTT compared to parental J82

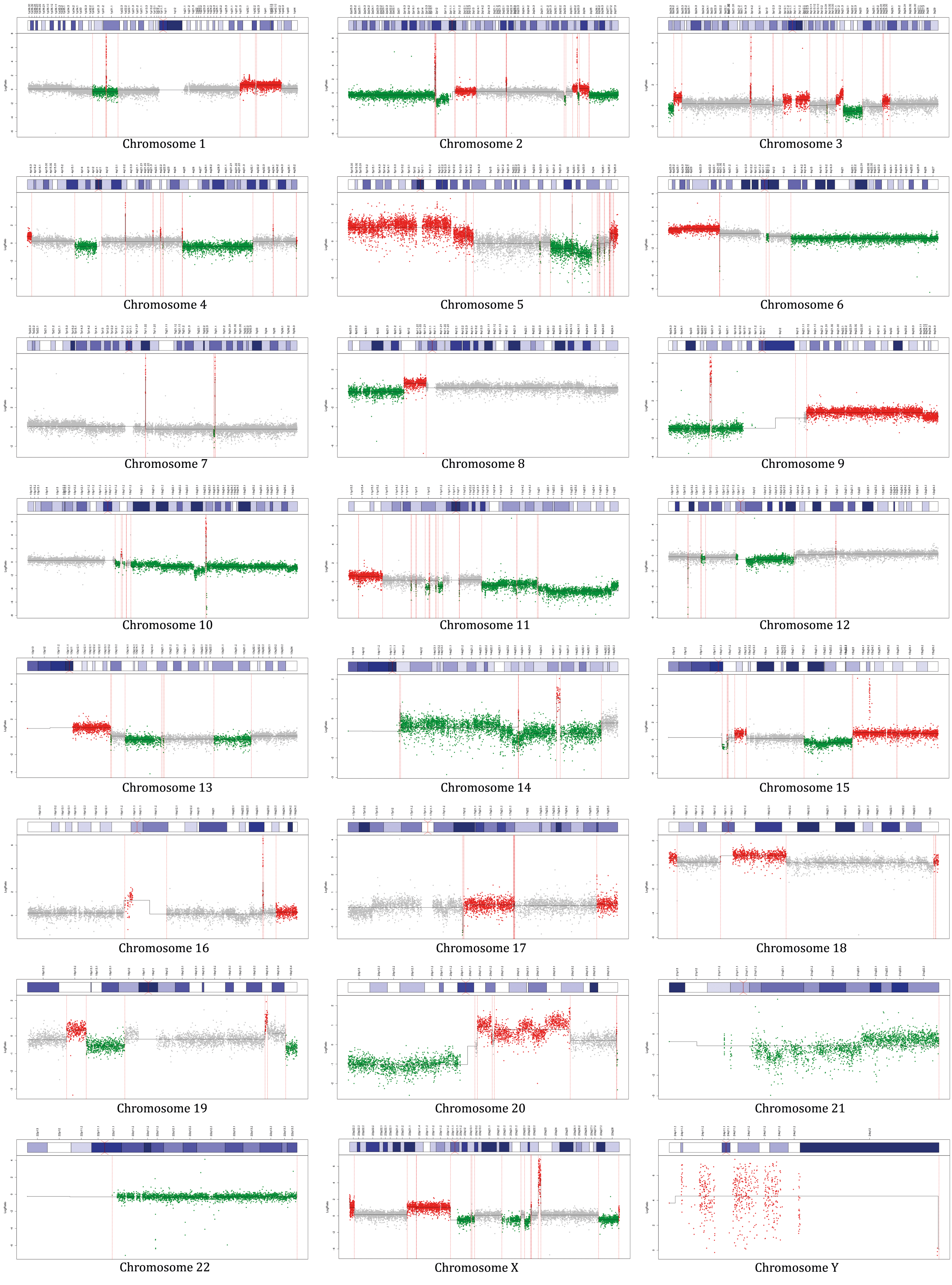

Supplementary figure 2

253J-LTT compared to parental 253J

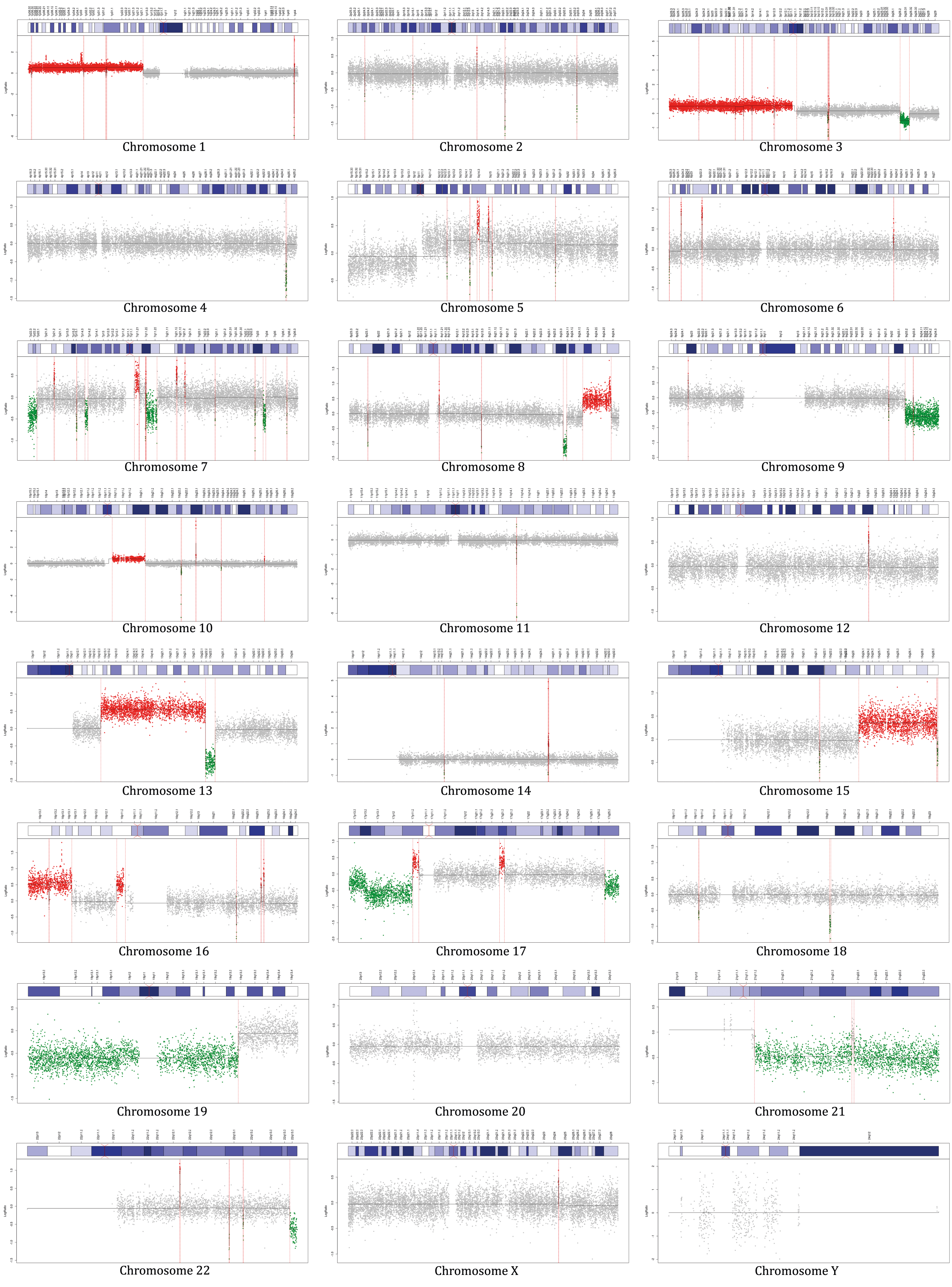

Supplementary figure 3

T-24-LTT compared to parental T-24

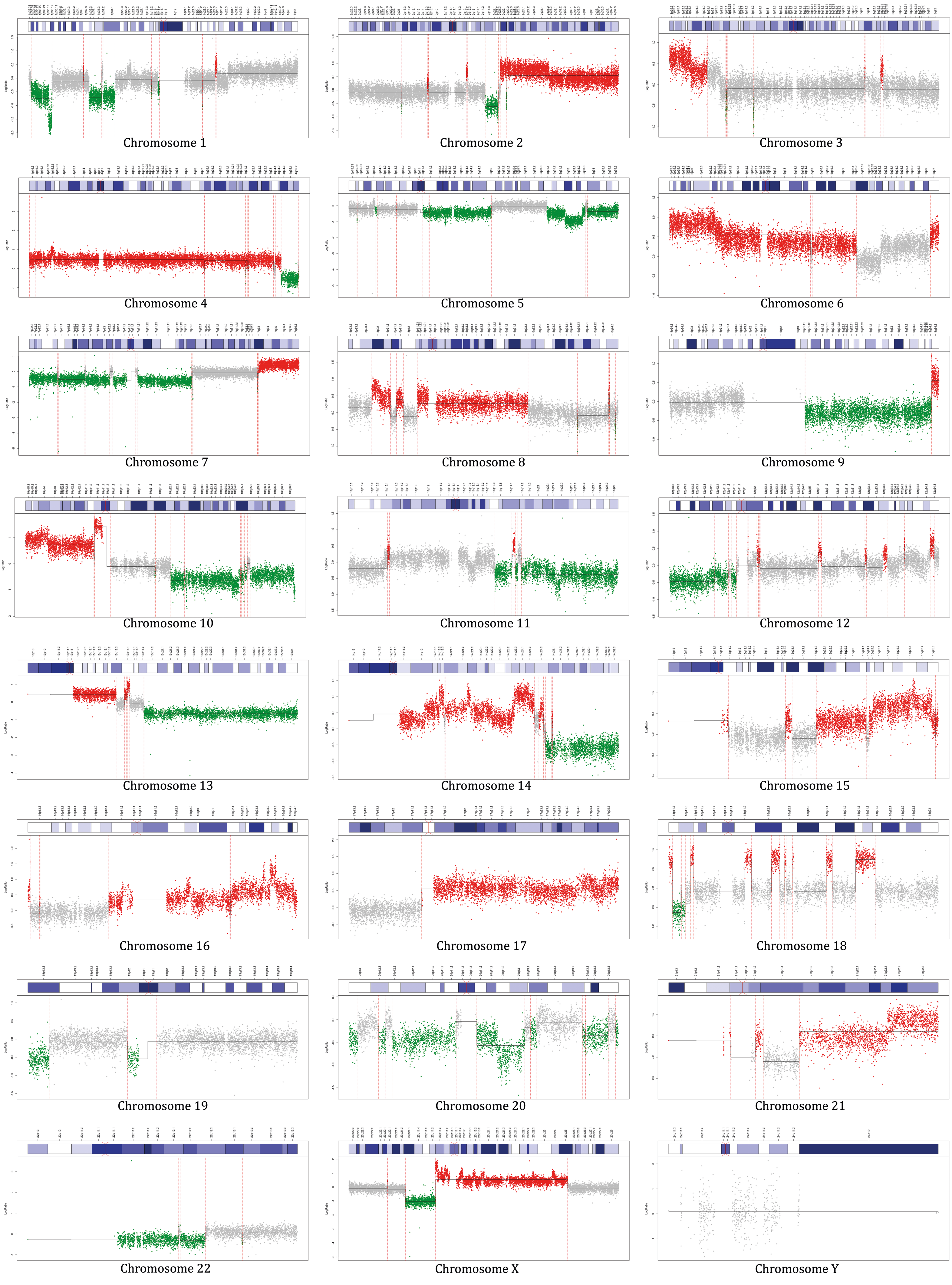

Supplementary figure 4a

Parental RT-112 and RT-112-LTT compared to pool Chr 1 - 12

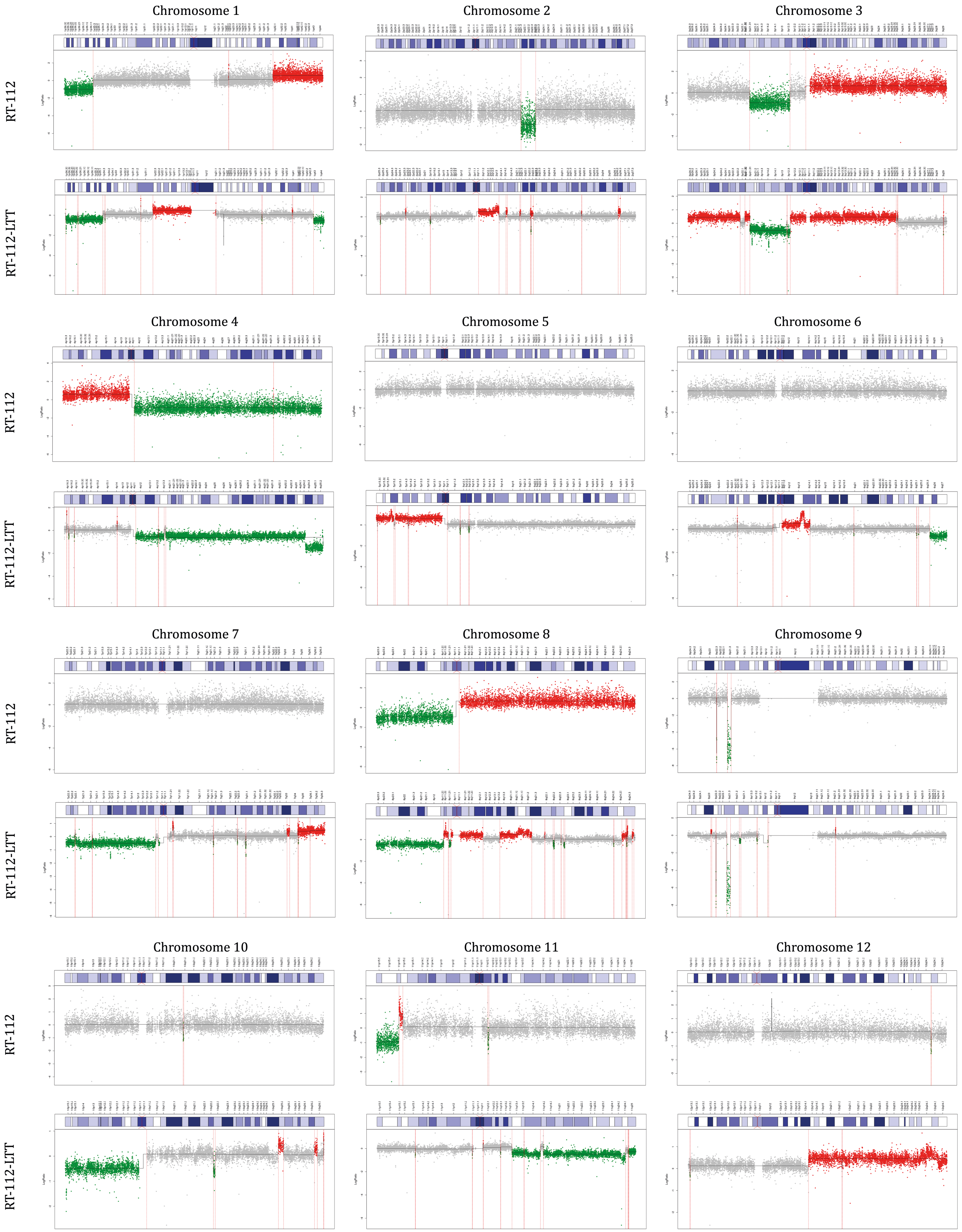

Supplementary figure 4b

Parental RT-112 and RT-112-LTT compared to pool Chr 13 - 24

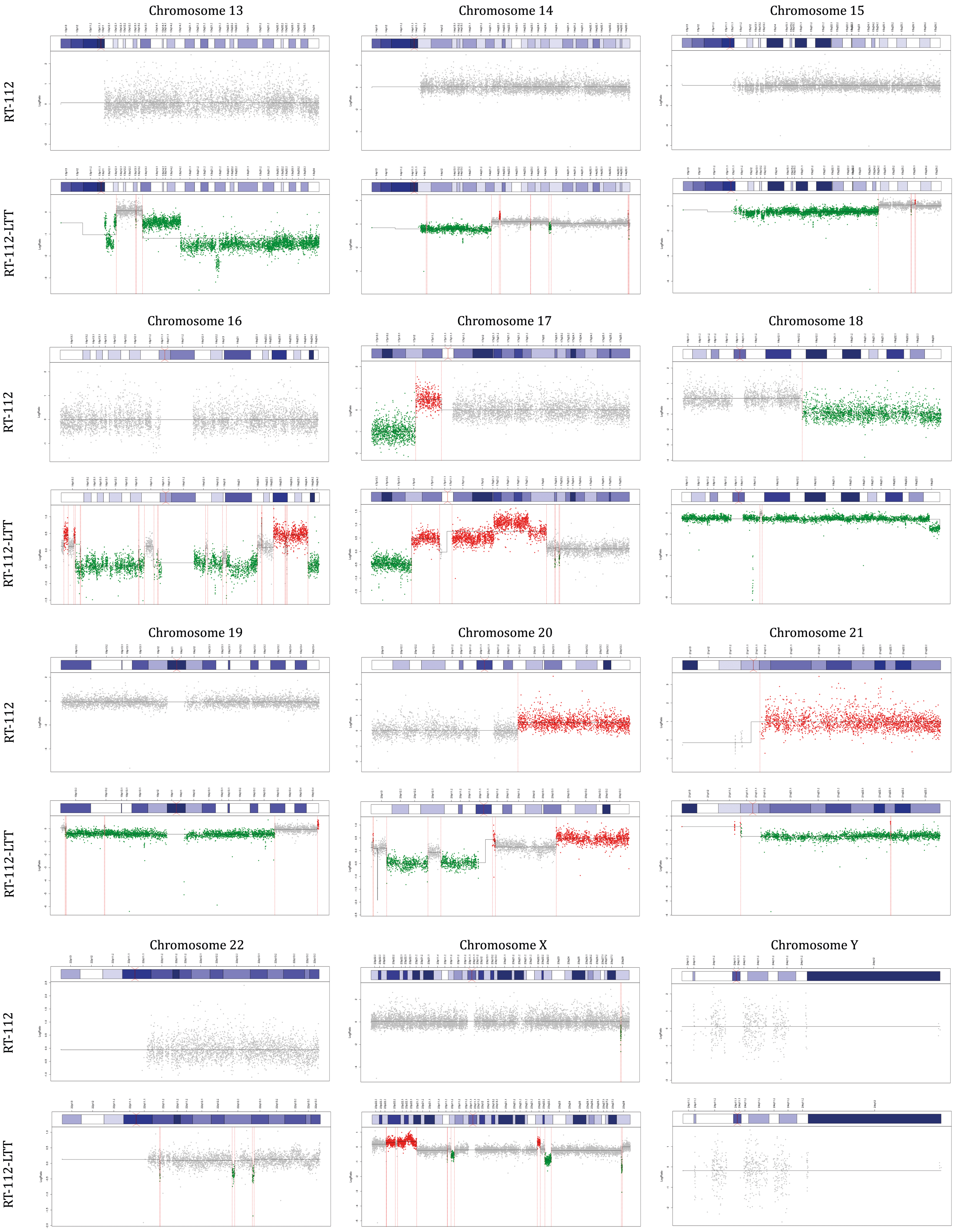

Supplementary figure 5

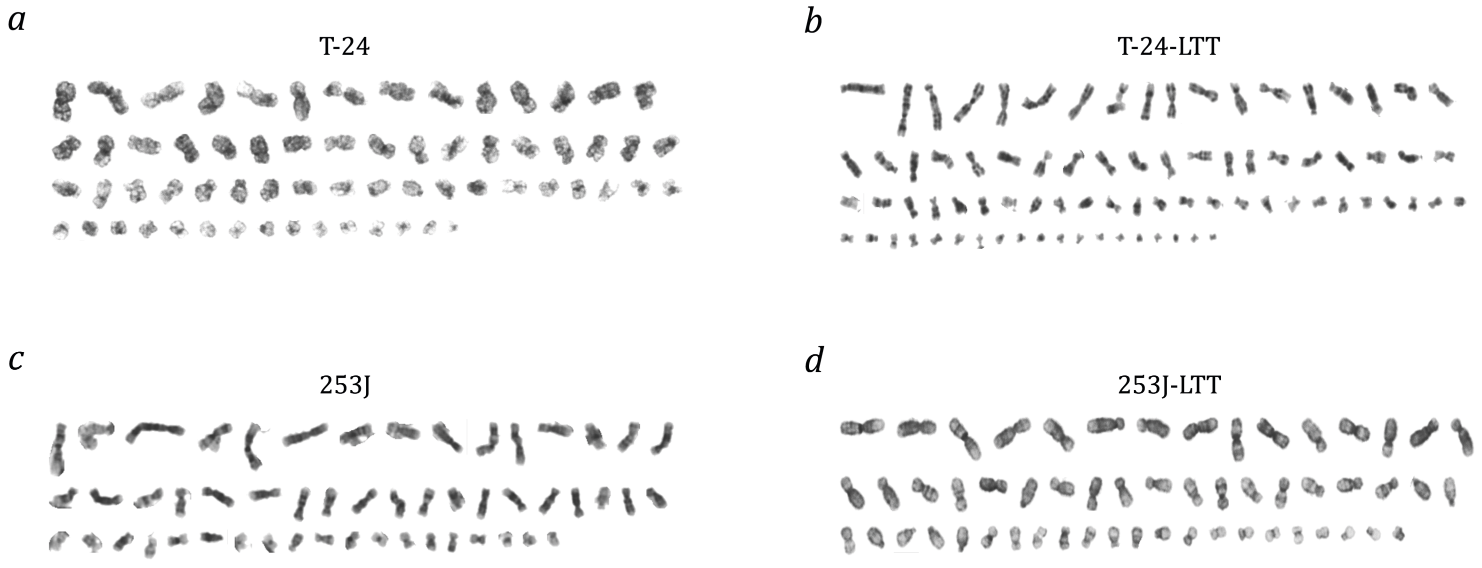

Supplementary figure 6a

RT-112-LTT compared to parental RT-112 T1 - T5 Chromosomes 1 - 6

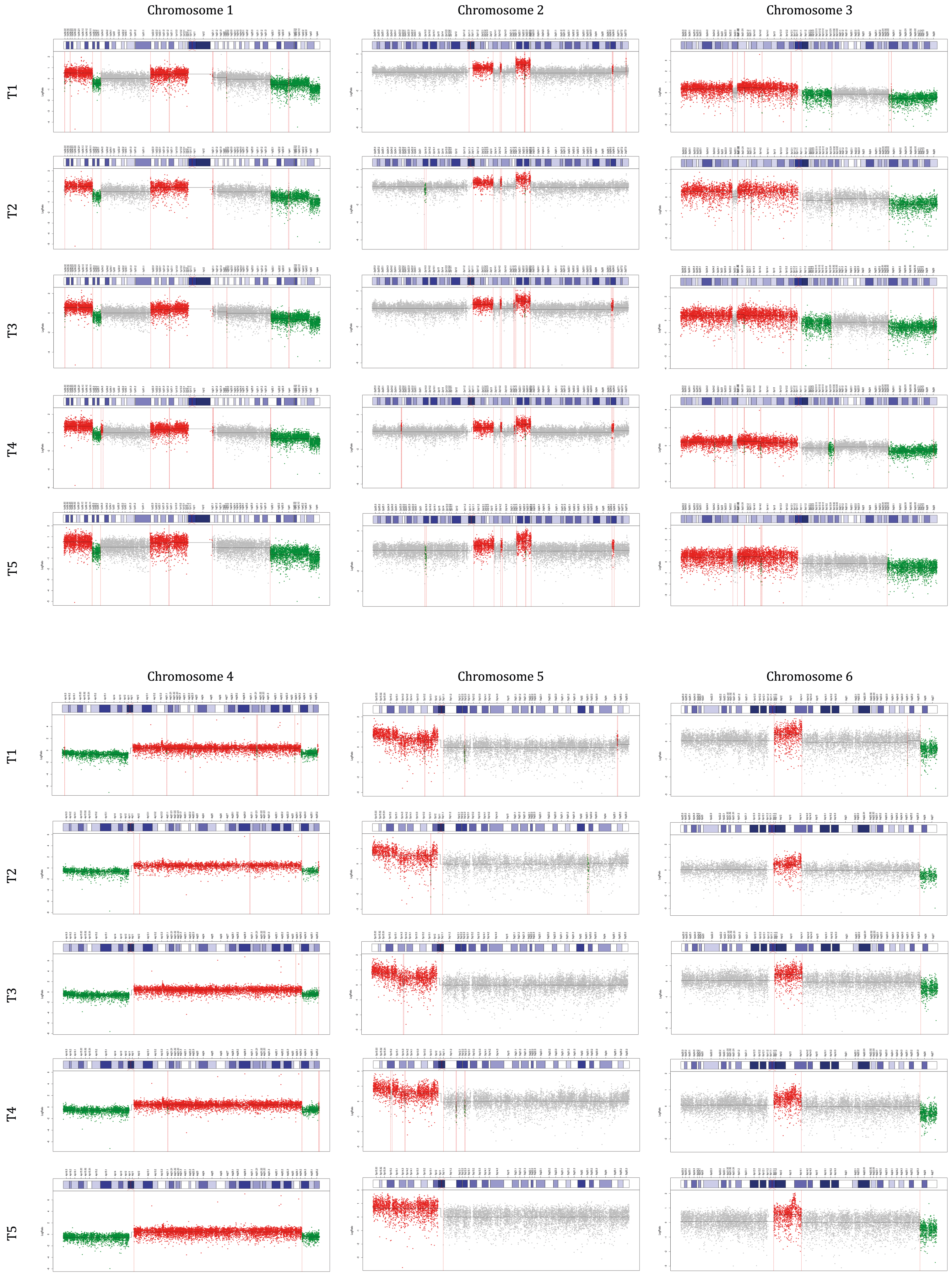

Supplementary figure 6b

RT-112-LTT compared to parental RT-112 T1 - T5 Chromosomes 7 - 12

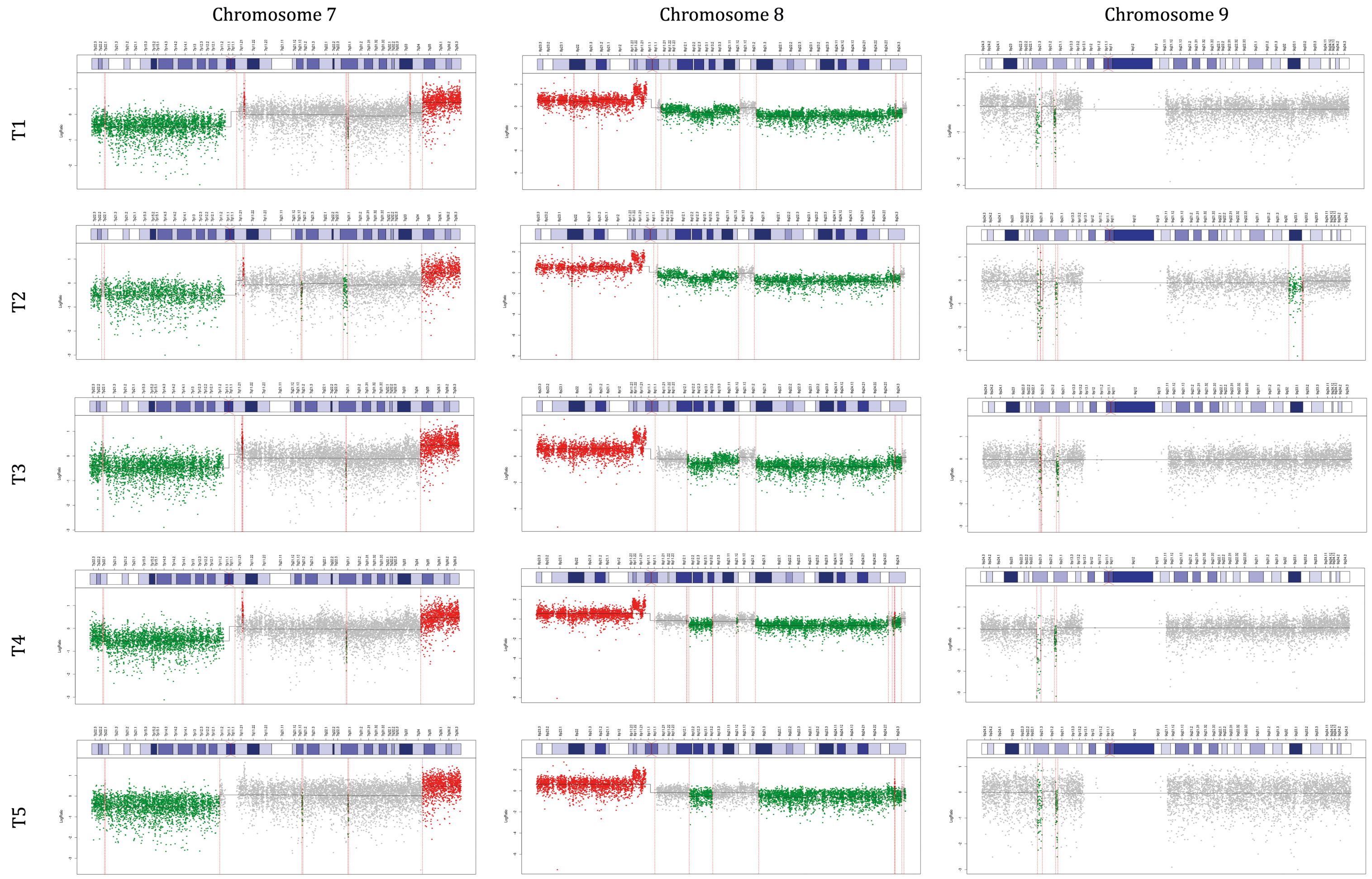

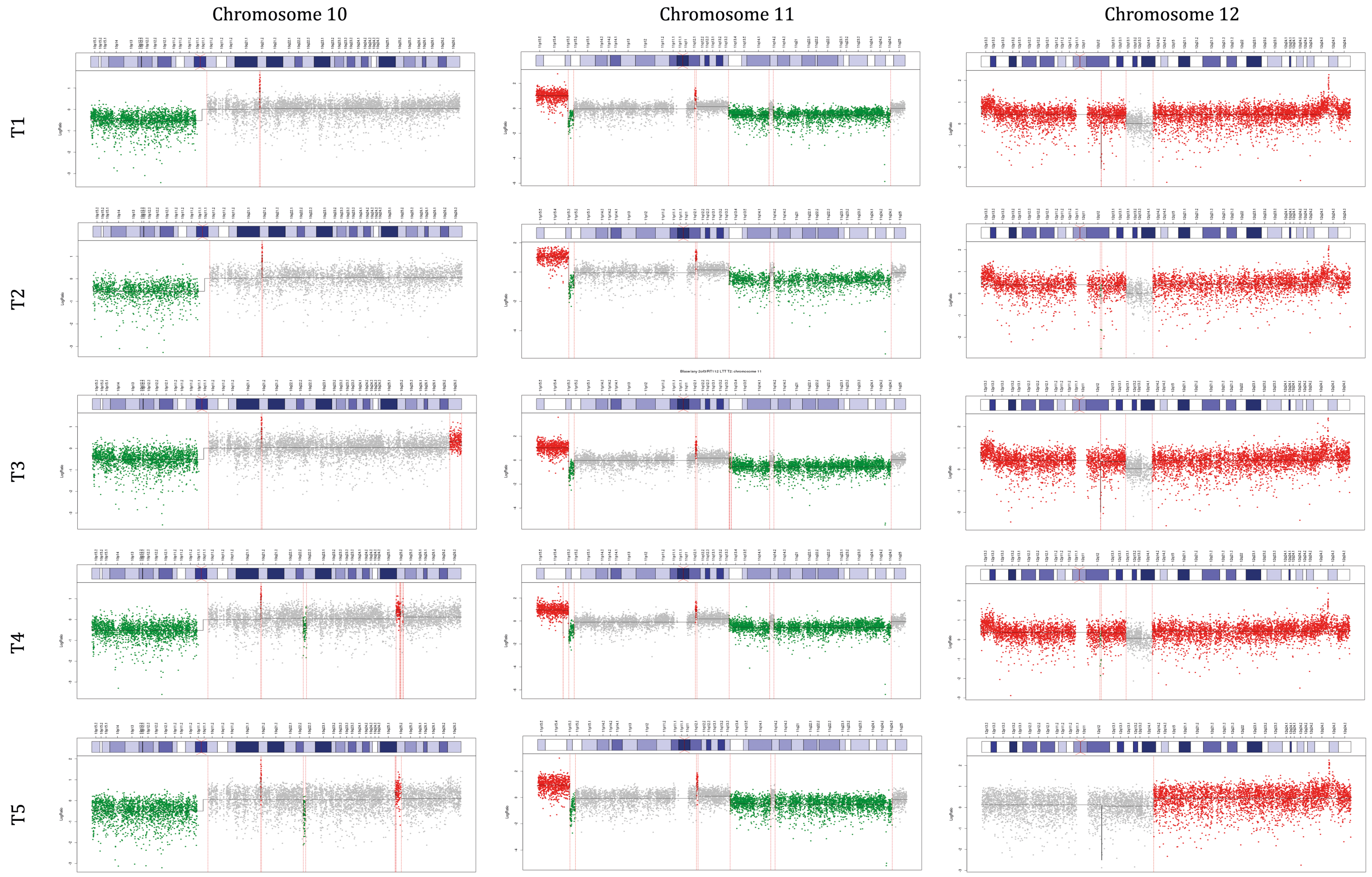

Supplementary figure 6c

RT-112-LTT compared to parental RT-112 T1 - T5 Chromosomes 13 - 18

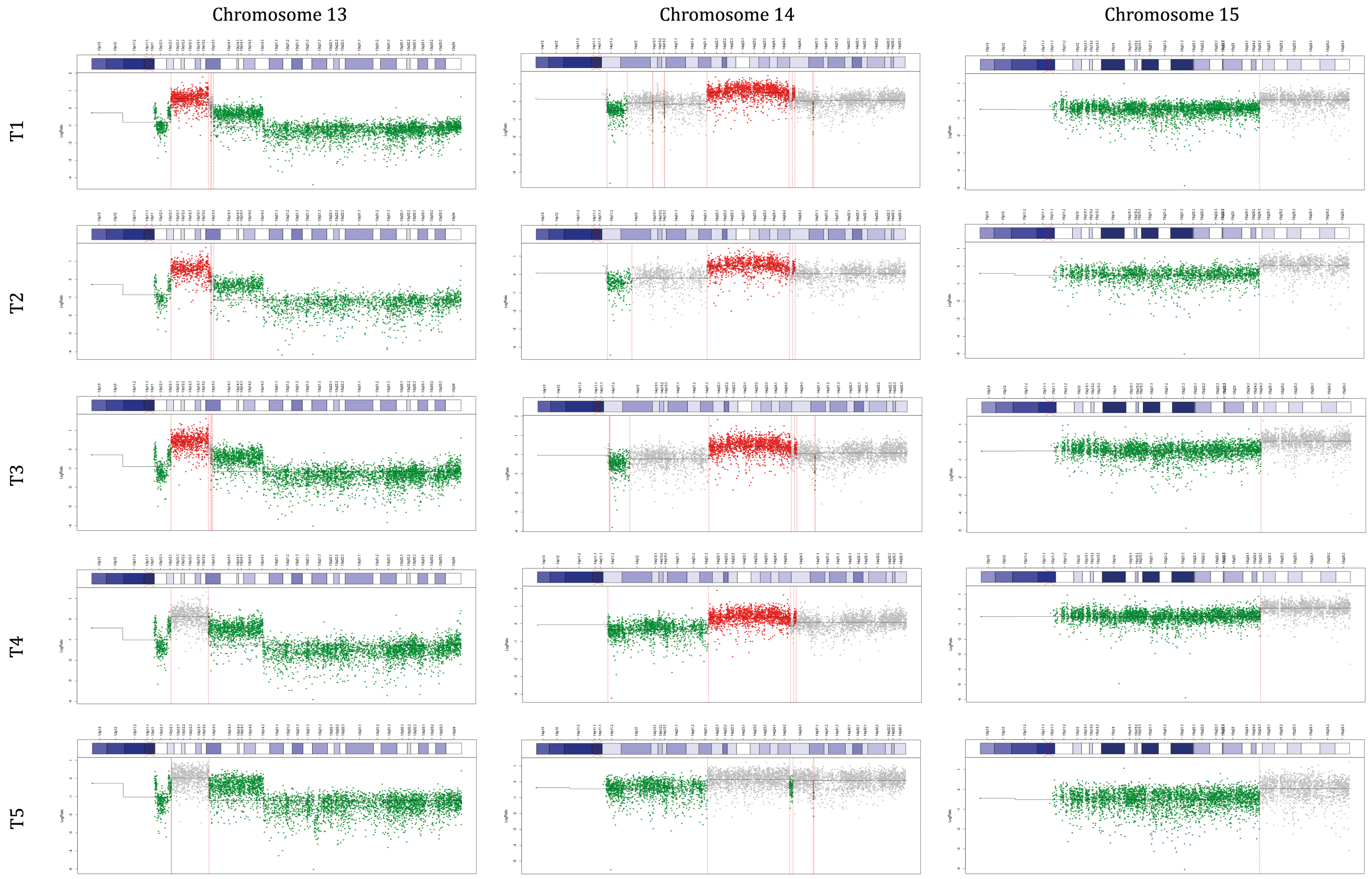

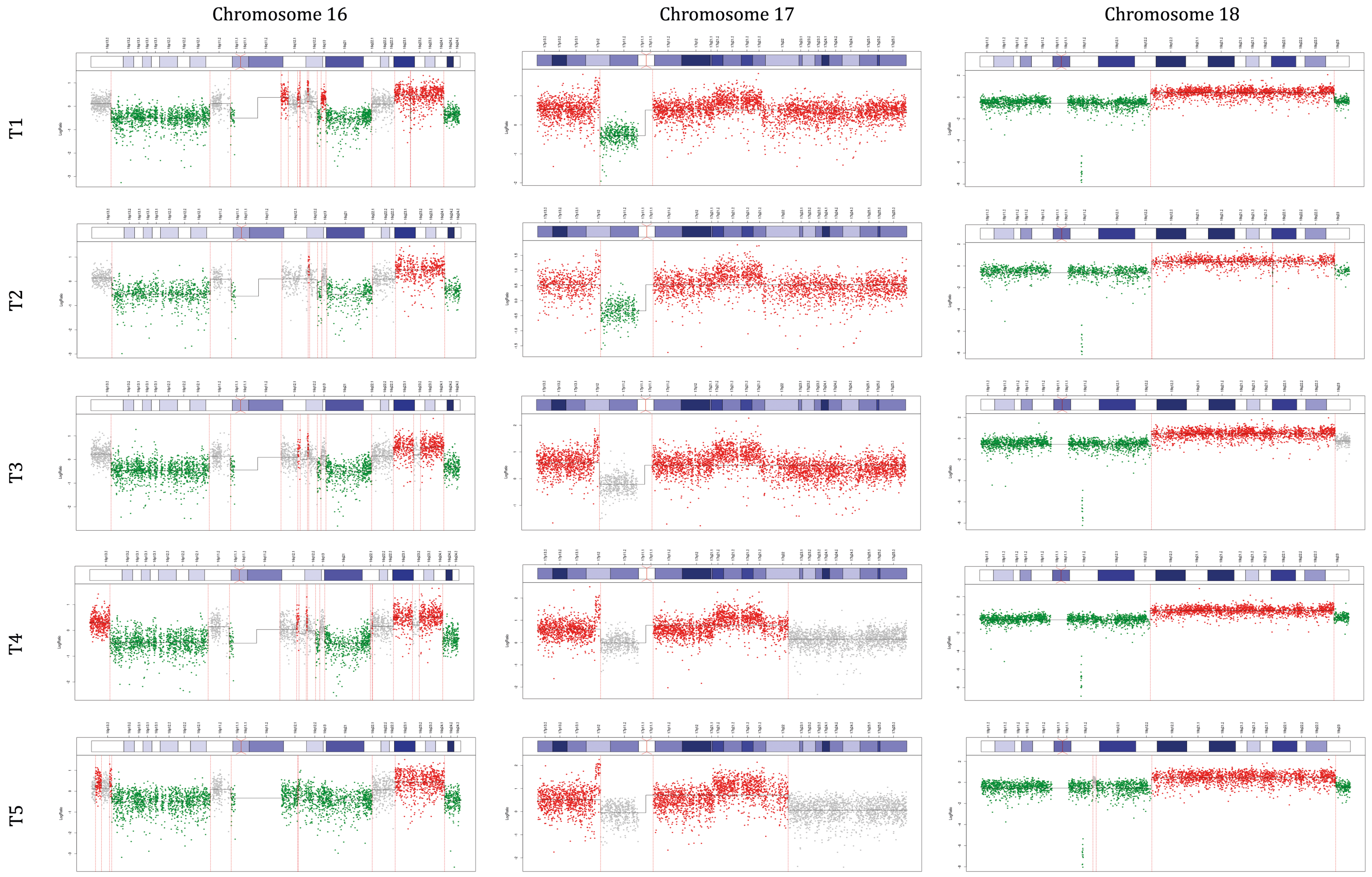

Supplementary figure 6d

RT-112-LTT compared to parental RT-112 T1 - T5 Chromosomes 19 - 24

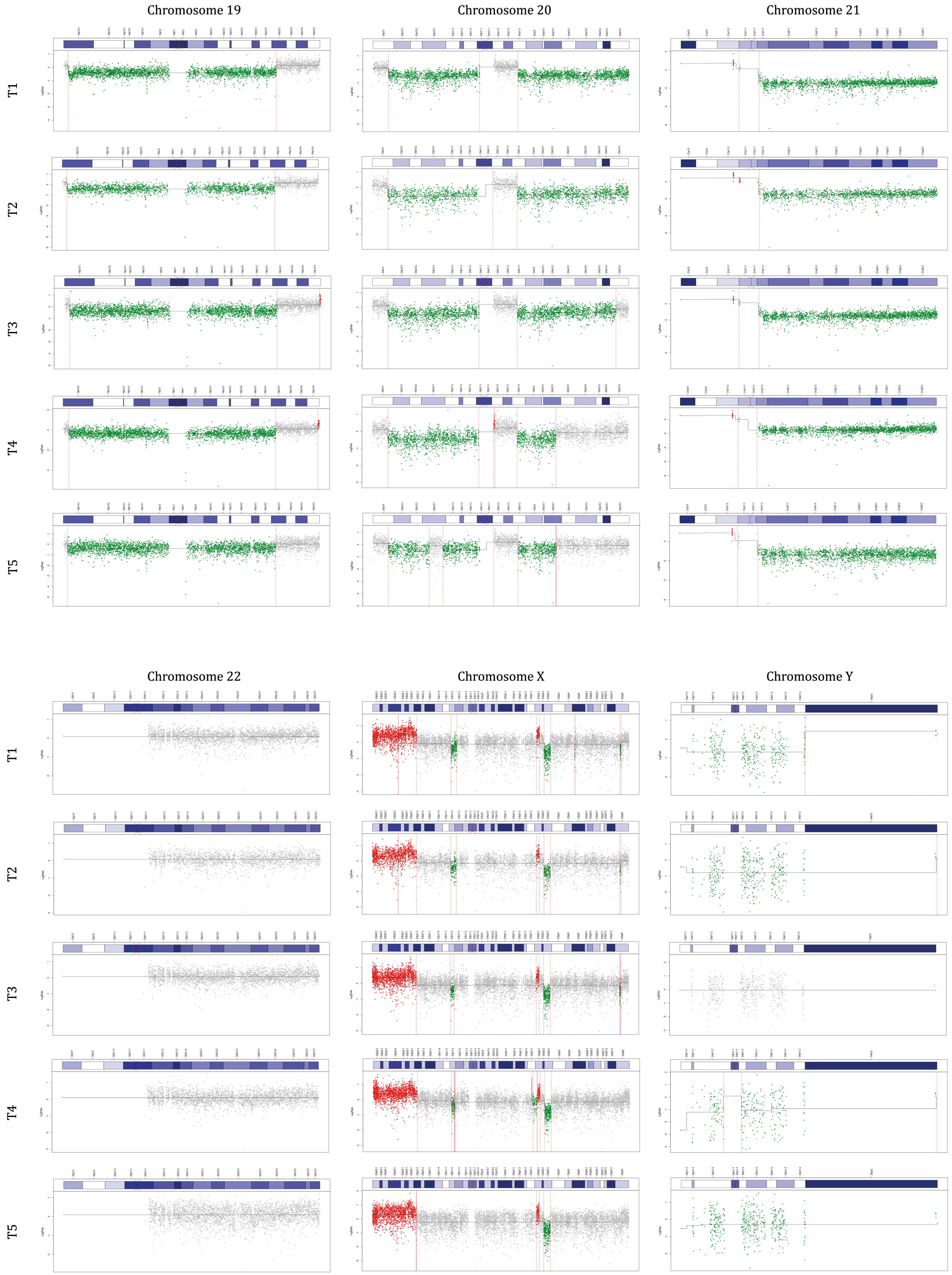

Supplementary figure 7a

J82-LTT compared to parental J82 T1 - T5 Chromosomes 1 - 6

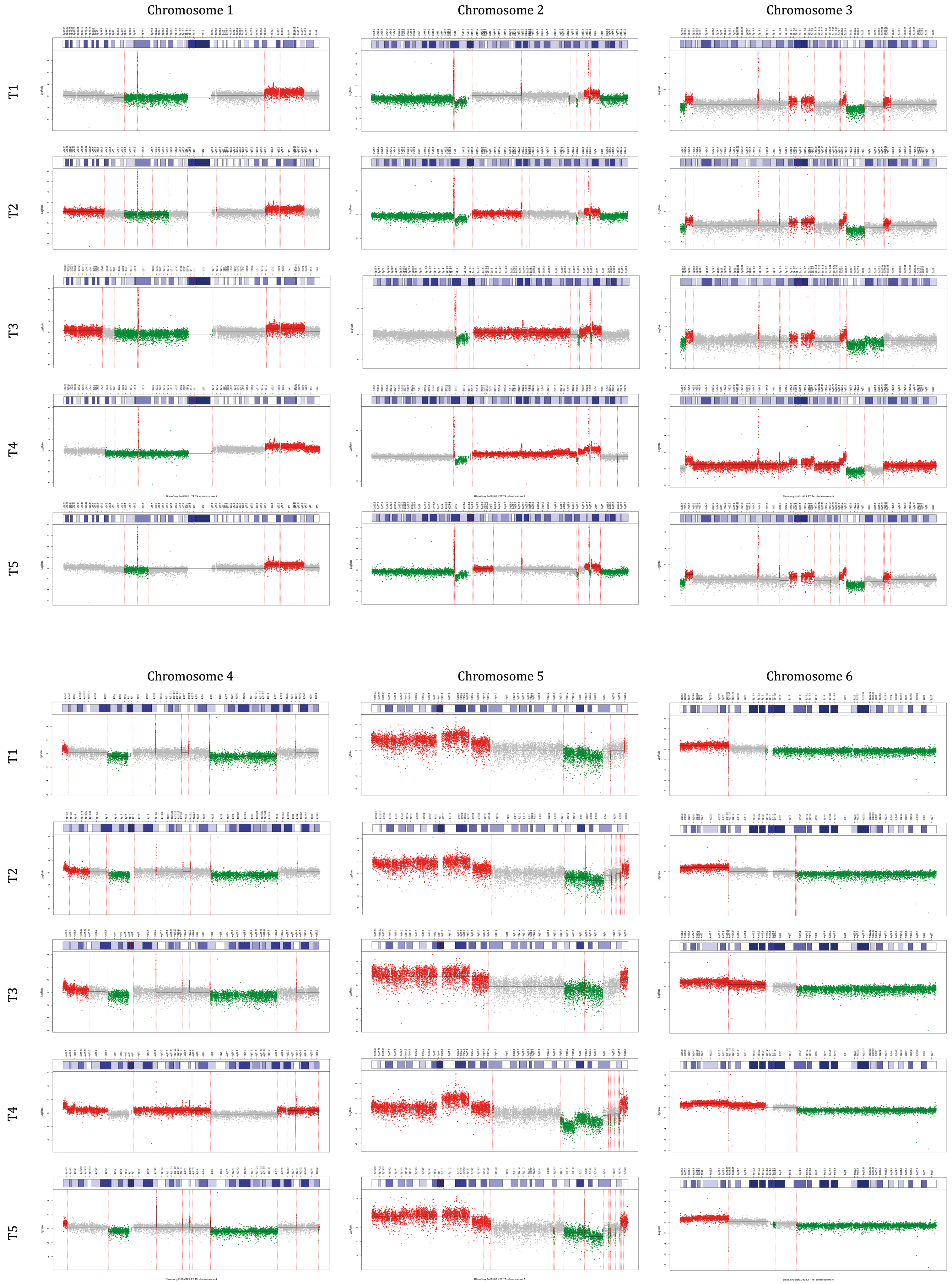

Supplementary figure 7b

J82-LTT compared to parental J82 T1 - T5 Chromosomes 7 - 12

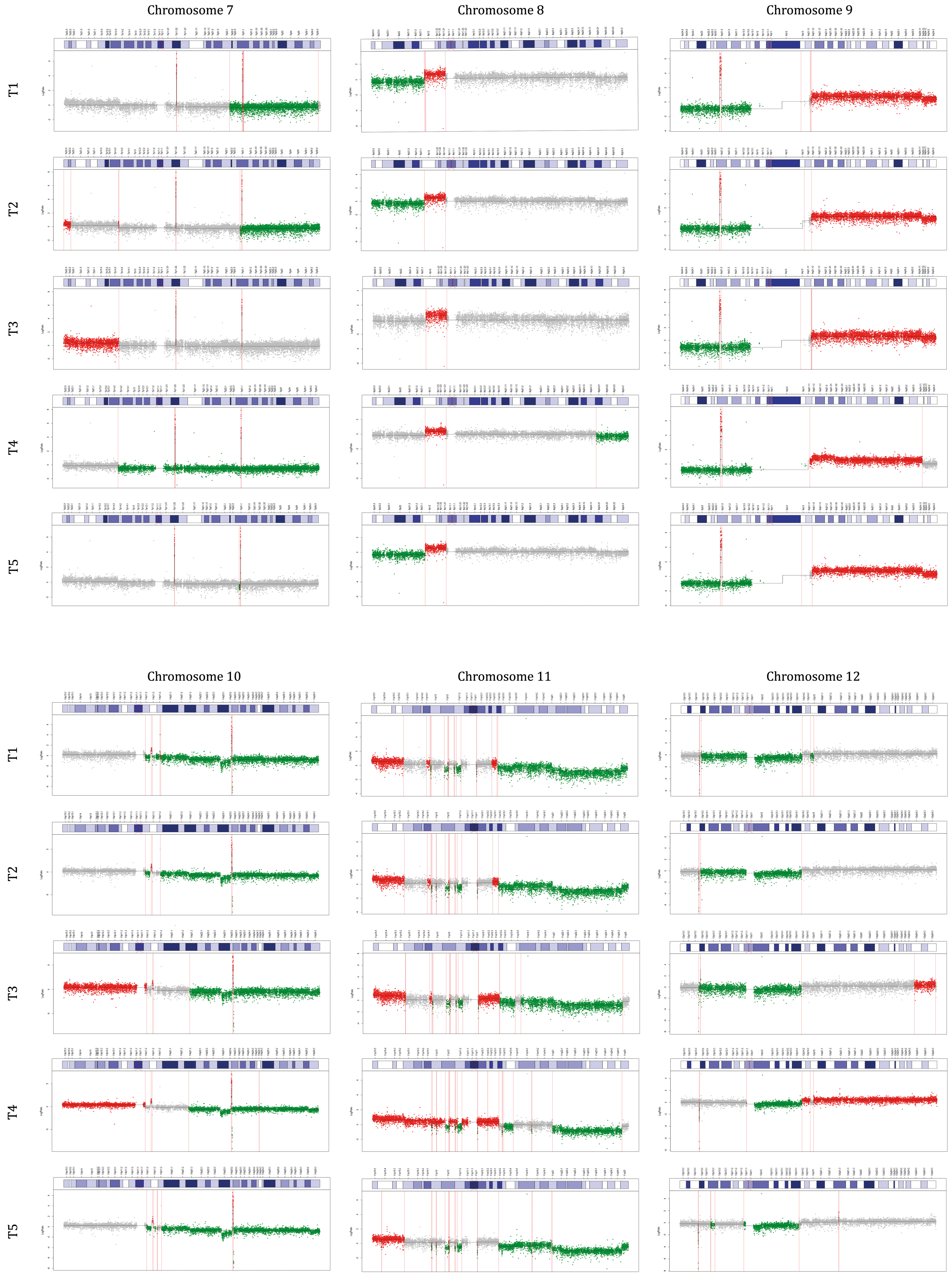

Supplementary figure 7c

J82-LTT compared to parental J82 T1 - T5 Chromosomes 13 - 18

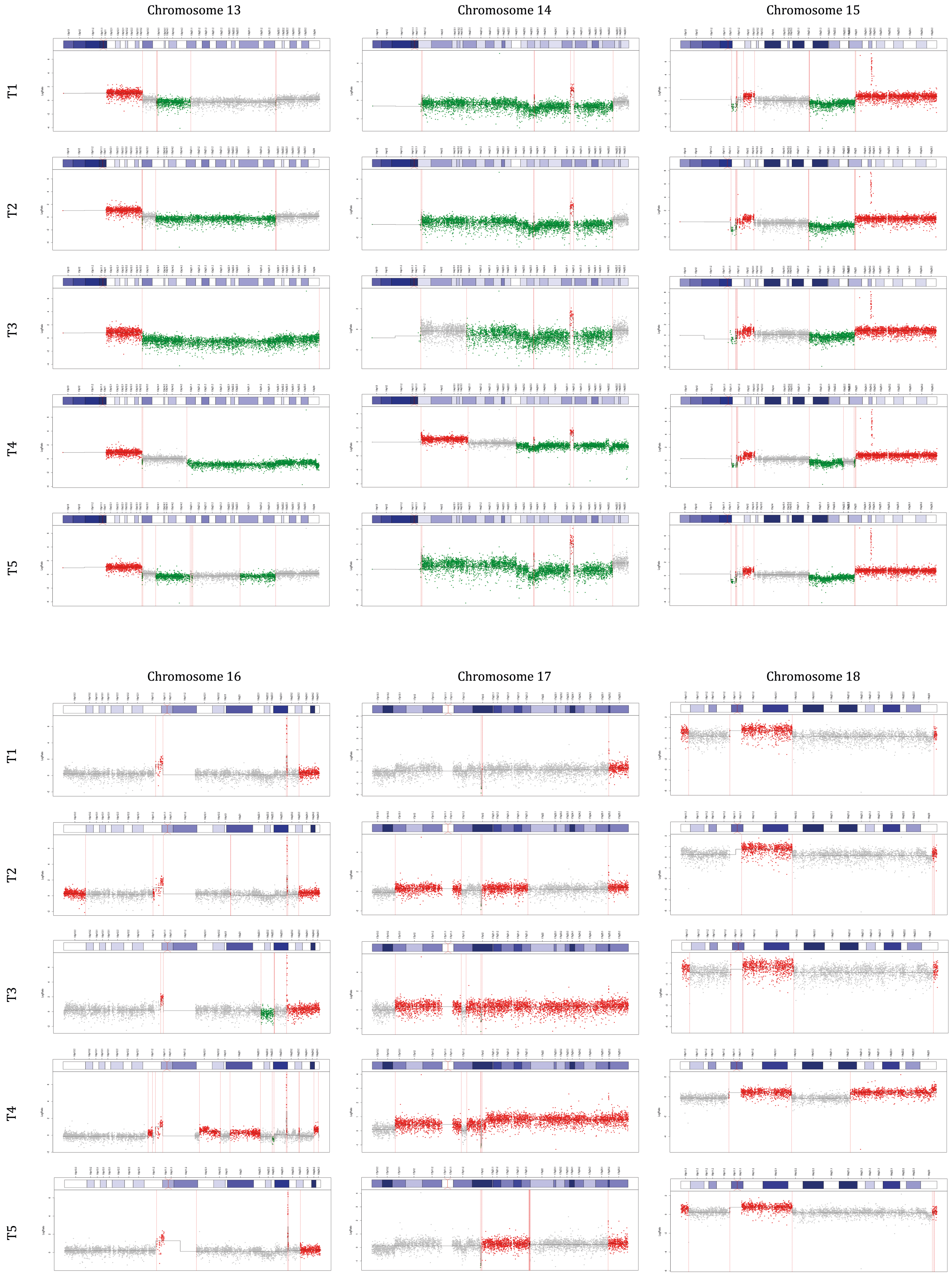

Supplementary figure 7d

J82-LTT compared to parental J82 T1 - T5 Chromosomes 19 - 24

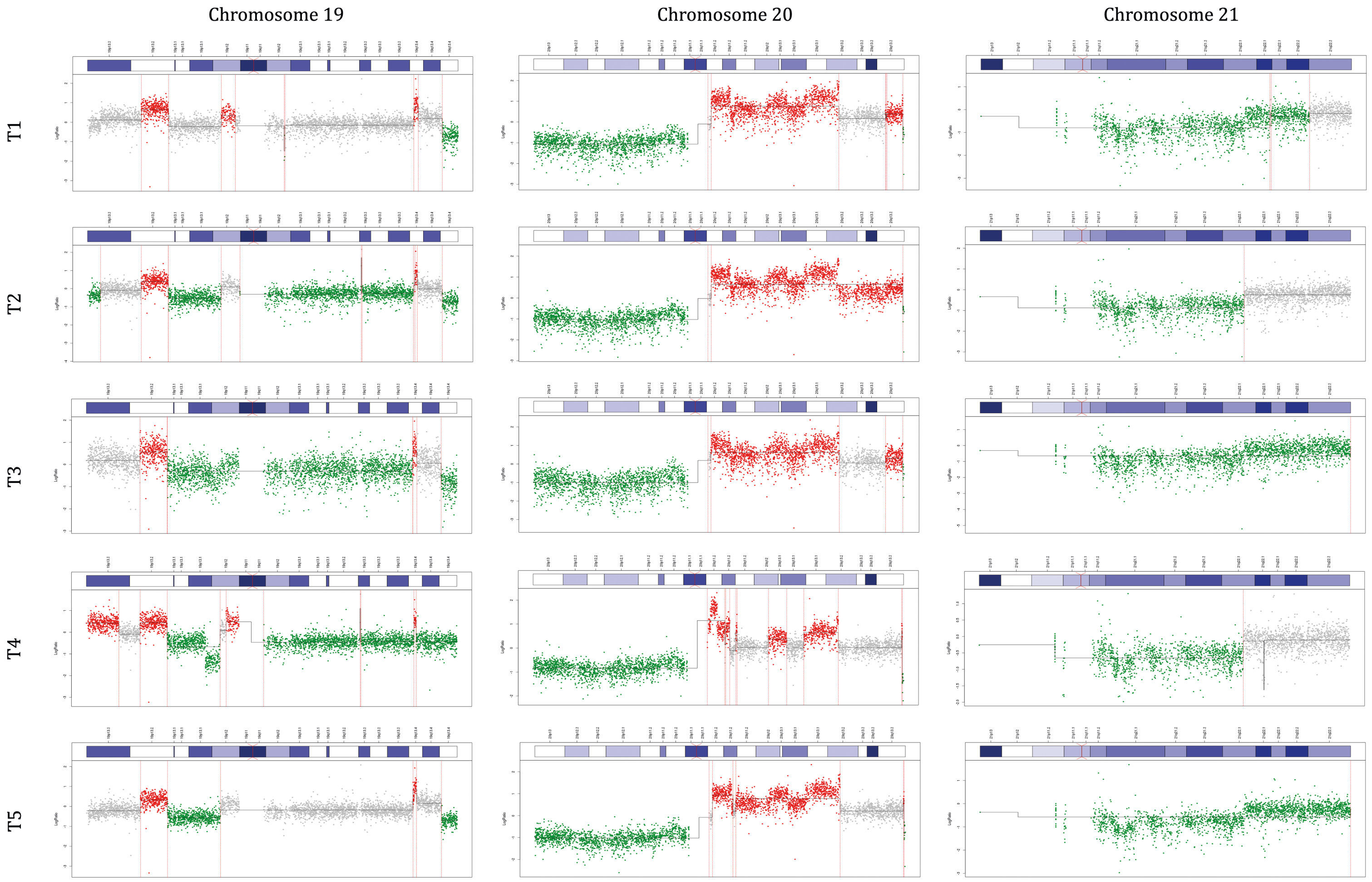

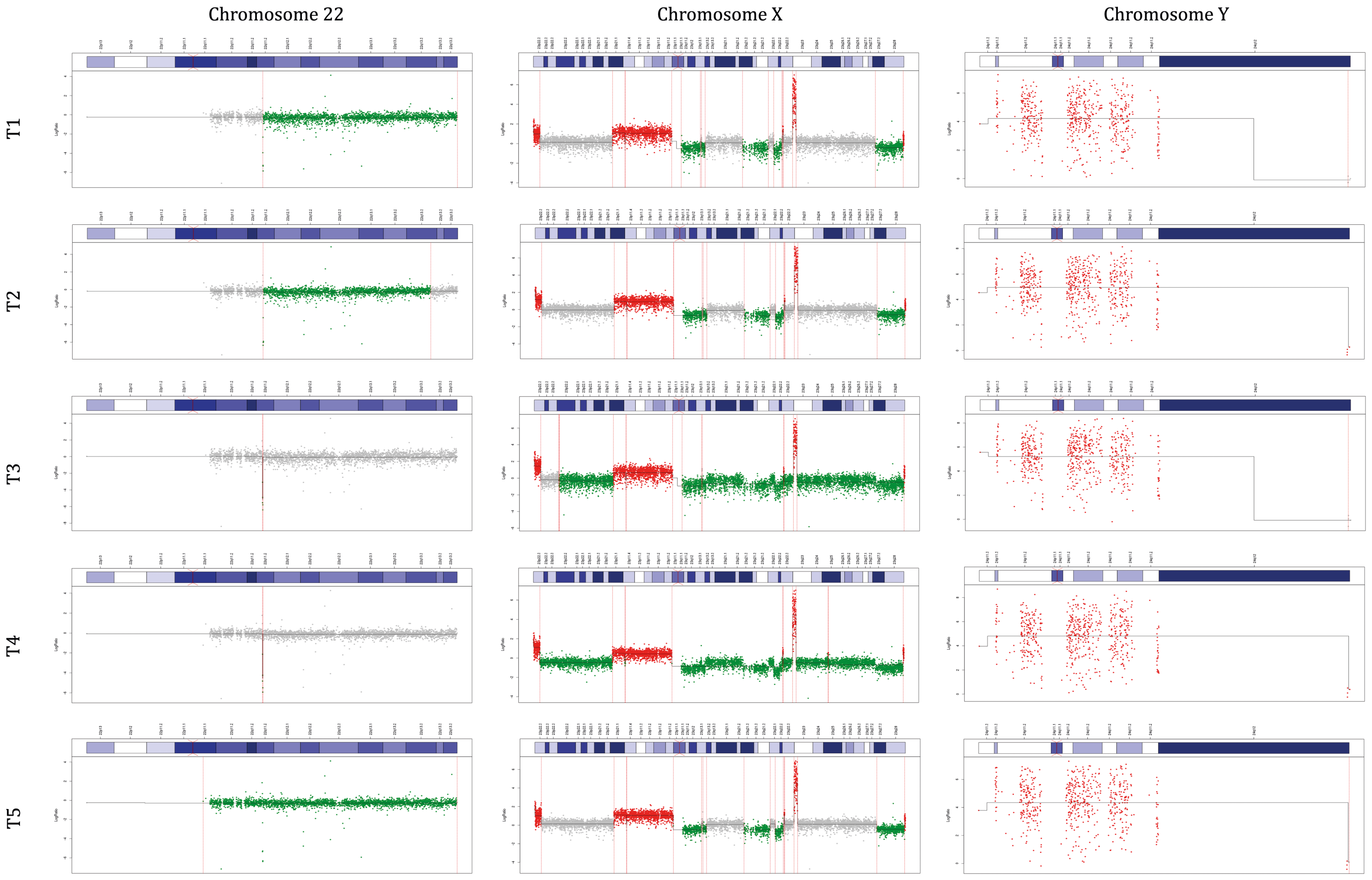

Supplementary Figure Legends

Figure S1. Chromosomal alterations of J82-LTT hybridized against their parental cell line as detected for each chromosome by array CGH.

Figure S2. Chromosomal alterations of 253J-TT hybridized against their parental cell line as detected for each chromosome by array CGH.

Figure S3. Chromosomal alterations of T-24-LTT hybridized against their parental cell line as detected for each chromosome by array CGH.

Figure S4. Chromosomal alterations of RT-112 and RT-112-LTT hybridized against pool of normal controls for chromosomes a) 1 – 12 and b) 13 – X) as detected by array CGH.

Figure S5. Massive genomic changes in LTTs. Karyotype of a) T-24, b) T-24-LTT, c) 253J, and d) 253J-LTT as detected by standard Giemsa staining.

Figure S6. Chromosomal alterations of RT-112-LTT at five different timepoints (T1 – T5) hybridized against their parental cell line for chromosomes a) 1 – 6, b) 7 – 12, c) 8 – 18, and d) 19 – X) as detected by array CGH.

Figure S7. Chromosomal alterations of J82-LTT at five different timepoints (T1 – T5) hybridized against their parental cell line for chromosomes a) 1 – 6, b) 7 – 12, c) 8 – 18, and d) 19 – Y) as detected by array CGH.

Supplementary Tables Legends

Table S1: All variants listed individually for RT-112 compared to the hg19 reference genome. Top gene candidates that are involved in cisplatin resistance or are mutated in the TCGA dataset are listed in the second and third tab, respectively.

Table S2: All variants listed individually for RT-112-LTT compared to the hg19 reference genome. Top gene candidates that are involved in cisplatin resistance or are mutated in the TCGA dataset are listed in the second and third tab, respectively.

Table S3: All variants listed individually for J82 compared to the hg19 reference genome. Top gene candidates that are involved in cisplatin resistance or are mutated in the TCGA dataset are listed in the second and third tab, respectively.

Table S4: All variants listed individually for J82-LTT compared to the hg19 reference genome. Top gene candidates that are involved in cisplatin resistance or are mutated in the TCGA dataset are listed in the second and third tab, respectively.

Table S5: All variants listed individually for 253 compared to the hg19 reference genome. Top gene candidates that are involved in cisplatin resistance or are mutated in the TCGA dataset are listed in the second and third tab, respectively.

Table S6: All variants listed individually for 253J-LTT compared to the hg19 reference genome. Top gene candidates that are involved in cisplatin resistance or are mutated in the TCGA dataset are listed in the second and third tab, respectively.

Table S7: All variants listed individually for T-24 compared to the hg19 reference genome. Top gene candidates that are involved in cisplatin resistance or are mutated in the TCGA dataset are listed in the second and third tab, respectively.

Table S8: All variants listed individually for T-24-LTT compared to the hg19 reference genome. Top gene candidates that are involved in cisplatin resistance or are mutated in the TCGA dataset are listed in the second and third tab, respectively.

Table S9: Individually listed variants that are uniquely altered in RT-112-LTT compared to their parental cell line. Top gene candidates that are involved in cisplatin resistance or are mutated in the TCGA dataset are listed in the second and third tab, respectively.

Table S10: Individually listed variants that are uniquely altered in J82-LTT compared to their parental cell line. Top gene candidates that are involved in cisplatin resistance or are mutated in the TCGA dataset are listed in the second and third tab, respectively.

Table S11: Individually listed variants that are uniquely altered in 253J-LTT compared to their parental cell line. Top gene candidates that are involved in cisplatin resistance or are mutated in the TCGA dataset are listed in the second and third tab, respectively.

Table S12: Individually listed variants that are uniquely altered in T-24-LTT compared to their parental cell line. Top gene candidates that are involved in cisplatin resistance or are mutated in the TCGA dataset are listed in the second and third tab, respectively.

Table S13: Summary of unique variants involved in cisplatin resistance and found in TCGA SMG in LTT sublines as detected by exome sequencing.

Table S14: Summary of commonly mutated genes in LTT sublines as detected by exome sequencing.

Table S15: Chromosomal alterations, gains, losses, and involved genes in RT-112-LTT, J82-LTT, 253J-LTT, and T-24-LTT sublines compared to their parental cell lines for each chromosome (Tabs 1 – 24) and alterations found in at least three LTT sublines (Tab 25) as detected by array CGH.

Table S16: Chromosomal alterations, gains, losses, and involved genes in RT-112 and RT-112-LTT compared to a pool of normal controls for each chromosome (Tabs 1 – 24) and alterations found only in RT-112-LTT (Tab 25) as detected by array CGH.

Table S17: Chromosomal alterations, gains, losses, and involved genes in RT-112-LTT subline compared to their parental cell line for each chromosome (Tabs 1 – 24) at five different timepoints (T1 – T5) as detected by array CGH. Common alterations found in 2-4, 2-3, ≥4, and 5 time points are listed in tabs 25, 26, 27 and 28, respectively.

Table S18: Chromosomal alterations, gains, losses, and involved genes in J82-LTT subline compared to their parental cell line for each chromosome (Tabs 1 – 24) at five different timepoints (T1 – T5) as detected by array CGH. Common alterations found in 2-4, 2-3, ≥4, and 5 time points are listed in tabs 25, 26, 27 and 28, respectively.

Table S19: Details on sequencing procedure, filtering, and number of variants in RT-112, RT-112-LTT, J82, J82-LTT, 253J, 253J-LTT, T-24, and T-24-LTT.

Table S20: Raw signature data of cosine similarities among the mutational profiles of the LTT lines.
